# Comprehensive characterization of AP-1 adaptor complex genes in lung cancer reveals AP1AR as a novel prognostic and therapeutic biomarker

**DOI:** 10.7150/jca.125763

**Published:** 2026-01-01

**Authors:** Dahlak Daniel Solomon, I-Jeng Yeh, Hsin-Liang Liu, Che-Yu Su, Yung-Kuo Lee, Ching-Chung Ko, Hui-Ru Lin, Sachin Kumar, Do Thi Minh Xuan, Neethu Palekkode, Ayman Fathima, Hung-Yun Lin, Chih-Yang Wang, Meng-Chi Yen

**Affiliations:** 1 Graduate Institute of Cancer Biology and Drug Discovery, College of Medical Science and Technology, Taipei Medical University, Taipei 11031, Taiwan.; 2 Yogananda School of AI Computers and Data Sciences, Shoolini University, Solan 173229, India.; 3 Department of Emergency Medicine, Kaohsiung Medical University Hospital, Kaohsiung Medical University, Kaohsiung 80708, Taiwan.; 4 Graduate Institute of Clinical Medicine, College of Medicine, Kaohsiung Medical University, Kaohsiung 80708, Taiwan.; 5 Medical Laboratory, Medical Education and Research Center, Kaohsiung Armed Forces General Hospital, Kaohsiung 80284, Taiwan.; 6 Division of Experimental Surgery Center, Department of Surgery, Tri-Service General Hospital, National Defense Medical University, Taipei, 11490, Taiwan.; 7 Institute of Medical Science and Technology, National Sun Yat-Sen University, Kaohsiung, 80424, Taiwan.; 8 Department of Medical Imaging, Chi-Mei Medical Center, Tainan, Taiwan.; 9 Department of Health and Nutrition, Chia Nan University of Pharmacy and Science, Tainan, Taiwan.; 10 School of Medicine, College of Medicine, National Sun Yat-Sen University, Kaohsiung, Taiwan.; 11 Nursing Department, Kaohsiung Armed Forces General Hospital, Kaohsiung 80284, Taiwan.; 12 Ph.D. Program for Cancer Molecular Biology and Drug Discovery, College of Medical Science, Taipei Medical University, Taipei 11031, Taiwan.; 13 Faculty of Applied Sciences and Biotechnology, Shoolini University of Biotechnology and Management Sciences, Himachal Pradesh, India.; 14 Faculty of Pharmacy, Van Lang University, 69/68 Dang Thuy Tram Street, Binh Loi Trung Ward, Ho Chi Minh City, 70000, Vietnam.; 15 Department of Biotechnology, Mother Teresa Women's University, Kodaikanal, Tamil Nadu, 624101, India.; 16 Computer Engineering with specialization in Artificial Intelligence and Machine Learning, Presidency University, Yelahanka, Bengaluru 560064 India.; 17 TMU Research Center of Cancer Translational Medicine, Taipei Medical University, Taipei 11031, Taiwan.; 18 Traditional Herbal Medicine Research Center of Taipei Medical University Hospital, Taipei Medical University, Taipei 11031, Taiwan.; 19 Cancer Center, Wan Fang Hospital, Taipei Medical University, Taipei 11031, Taiwan.; 20 Pharmaceutical Research Institute, Albany College of Pharmacy and Health Sciences, Rensselaer, NY 12144, USA.

**Keywords:** AP-1 adaptor complex, AP1AR, lung cancer, biomarker, multi-omics analysis, single-cell RNA sequencing, prognosis and therapeutic target

## Abstract

Lung cancer remains the leading cause of cancer mortality. The AP-1 adaptor complex, including AP1AR, AP1S1, AP1S2, AP1S3, AP1M1, AP1M2, AP1B1, and AP1G1, functions as a conserved hub of vesicular trafficking, selecting cargo and coordinating clathrin-mediated transport. By shaping receptor recycling, membrane composition, and signal duration, AP-1 influences core cancer phenotypes such as proliferation, migration, and therapy response. However, the family-level role of AP-1 adaptors in lung cancer is incompletely defined. We systematically profiled all eight AP-1 adaptor genes using multi-omics datasets, survival resources, pharmacogenomic panels, Human Protein Atlas data, pathway enrichment, and single-cell RNA sequencing with cell-cell communication modeling. *AP1AR* was consistently upregulated in lung adenocarcinoma and independently associated with poorer overall survival. It was linked to cell-cycle progression, DNA replication checkpoints, hypoxia, and epithelial-to-mesenchymal transition (EMT). At single cell resolution, *AP1AR* also regulate malignant epithelial and fibroblast cell types. Pseudotime analyses revealed progressive activation along proliferative and EMT axes, and CellChat modeling indicated enhanced stromal and epithelial signaling. *AP1S3* and *AP1S1* showed complementary roles, associated with oncogenic/inflammatory signaling and immune-metabolic programs, respectively. These findings identify *AP1AR* as a clinically relevant biomarker and highlight AP-1 adaptor biology as an underexplored contributor to lung adenocarcinoma progression and therapeutic stratification.

## 1. Introduction

Lung cancer remains the leading cause of cancer-related mortality worldwide, with survival rates remaining poor despite advances in molecularly targeted therapies and immunotherapy 1, 2. Identifying novel molecular regulators of tumor progression is therefore essential for discovering new biomarkers and therapeutic targets 3-5. Non-small-cell lung cancer (NSCLC) accounts for approximately 85% of cases and is primarily divided into two major histological subtypes: lung adenocarcinoma (LUAD) and lung squamous cell carcinoma (LUSC). Adaptor protein (AP) complexes are evolutionarily conserved regulators of intracellular trafficking that mediate cargo sorting among endosomes, lysosomes, and the trans-Golgi network 6. Among them, the AP-1 adaptor complex plays a central role in clathrin-mediated transport, linking membrane dynamics to cell signaling and homeostasis 7. Dysregulation of AP complexes has been implicated in several cancers, where altered vesicle trafficking can affect oncogenic receptor turnover, nutrient signaling, and immune evasion 8-10. While several AP-1 adaptor subunits, such as *AP1B1* and *AP1G1*, have been studied in cancer 11, 12, a systematic evaluation of the AP-1 adaptor family in lung cancer is lacking. Notably, AP1AR (adaptor protein complex 1-associated regulatory) has not been characterized in lung cancer or other solid tumors, representing an opportunity to explore novel mechanisms of tumor regulation.

Recent multi-omics resources enable comprehensive evaluation of gene families across diverse cancer datasets 13-17. Integrative analyses combining bulk transcriptomics, clinical outcomes, protein expression, drug-sensitivity correlations, pathway enrichment, and single-cell data allow robust characterization of candidate genes 18-20. Using this approach, we present the first systematic analysis of eight AP-1 adaptor genes in lung cancer. We show that *AP1AR* is consistently upregulated, associated with poor survival, enriched for cell-cycle and epithelial-to-mesenchymal transition (EMT) pathways, and localized to malignant epithelial and fibroblast cell types at the single-cell level. *AP1S3* and *AP1S1* provide complementary support, while other family members offer broader context. These findings establish *AP1AR* as a novel prognostic biomarker and potential therapeutic target and highlight the AP-1 adaptor complex as an underexplored contributor to lung cancer biology.

## 2. Materials and Methods

### 2.1 RNA-seq Expression and Clinical Data

Transcriptomic expression data and corresponding clinical annotations for LUAD and normal lung tissues were obtained from UALCAN, which compiles The Cancer Genome Atlas (TCGA) Level 3 RNA-seq (HTSeq-FPKM) datasets 21, 22. Analyses included tumor versus regular comparisons, pathological stage-specific profiling, and pan-cancer assessments. Expression values were log2-transformed as transcripts per million (TPM + 1) to ensure comparability across datasets. Prognostic associations were evaluated using three independent platforms to enhance reproducibility 23-25. SurvivalGenie v2.0 was used to generate volcano plots and multivariate Cox regression-based forest plots across TCGA cohorts 26. GEPIA2 provided Kaplan-Meier survival curves and hazard ratios, while KMplotter was employed to validate associations in independent LUAD samples 27, 28. Patients were stratified into high- and low-expression groups based on the median value unless otherwise indicated 29-31. Statistical significance was determined using log-rank *p* values, with hazard ratios (HRs) and 95% confidence intervals (CIs) reported.

### 2.2 DNA-Methylation and Protein Expression Profiling

DNA-methylation data for *AP1AR* and *AP1S3* were obtained from the TCGA-LUAD and TCGA-LUSC cohorts via the UCSC Xena browser, and promoter-level β-values were analyzed 32. Methylation profiles were visualized using heatmaps and boxplots to compare tumor and adjacent normal tissues. Functional dependency data from DepMap (23Q4 release) were used to assess the essentiality of *AP1AR* and *AP1S3* across lung cancer cell lines 33. Protein expression and subcellular localization were evaluated using immunohistochemistry (IHC) data from the Human Protein Atlas (HPA) 34. Representative staining images for normal and tumor lung tissues were examined, and staining intensity was classified as not detected, low, medium, or high. Localization patterns were compared between tumor and normal specimens to validate transcriptomic observations at the protein level 35-37.

### 2.3 Drug-Sensitivity and Gene Set Enrichment and Pathway Analyses

Drug response correlations were analyzed through the Gene Set Cancer Analysis (GSCA) platform 38, supplemented with data from the Cancer Therapeutics Response Portal (CTRP) 39 and the Genomics of Drug Sensitivity in Cancer (GDSC) 40. Gene expression levels were correlated with half-maximal inhibitory concentration (IC_50_) values using Pearson or Spearman correlation coefficients. Associations with false discovery rate (FDR)-adjusted *p* values of < 0.05 were considered statistically significant 41-43. Functional enrichment analyses were performed using Gene Set Enrichment Analysis (GSEA) and MetaCore (Clarivate Analytics). GSEA was conducted utilizing the fgsea R package (Bioconductor v3.19) 44, which employs hallmark and curated gene sets from the Molecular Signatures Database (MSigDB v7.5.1) 45. Analyses were based on 10,000 permutations, and pathways with normalized enrichment scores (NESs) and an FDR of < 0.05 were considered significant. MetaCore was used to validate enrichment results and identify curated pathways relevant to cancer progression 46-48. Additionally, protein-protein interaction (PPI) networks among the eight AP-1 adaptor genes were generated using STRING v12.0, with a medium-confidence threshold set to 0.4 to identify functionally relevant interactions 49.

### 2.4 Single-Cell Transcriptomic and Cell-Cell Communication Analysis

Single-cell RNA-seq data were analyzed using the GSE202159 dataset 50, accessed via the cellxgene platform 51. Processed data, including quality-controlled cell clusters and annotated major lineages (epithelial, fibroblast, endothelial, myeloid, lymphoid, and T/natural killer (NK) cells), were used for downstream analyses. Gene expression patterns of the AP-1 adaptor genes were visualized using t-distributed stochastic neighbor embedding (t-SNE) dimensionality-reduction techniques. Expression intensities were displayed on feature plots, and relative enrichment was assessed across clusters via heatmaps 52-54. Cell-cycle states (G1, S, and G2/M) were inferred using canonical phase markers. Pseudotime trajectories were reconstructed using the Slingshot algorithm within the SingleCellPipeline (SCP) package to infer lineage relationships and temporal expression trends for *AP1AR* and *AP1S3*. Correlation analyses were performed against DNA-repair gene sets (*BRCA1*, *RAD51*, *ATM* and *PARP1).* Clinical metadata, including tumor stage, histological subtype, and smoking status, were integrated for contextual interpretation and analysis 55-58. Clinical metadata, including tumor stage, histological subtype, and smoking status, were incorporated to contextualize expression patterns within tumor progression. To evaluate intercellular signaling networks associated with target gene expressions, CellChat 59 was applied to the GSE202159 object. Separate analyses were performed for high- and low-expression cell subsets. The inferred signaling probabilities were visualized as global communication networks, lineage-specific connectivity heatmaps, and directional sender-receiver maps 60-64.

### 2.5 Statistical Analysis

All analyses were performed using publicly available platforms and locally installed software 65-67. Data handling and visualization used R/RStudio with the ggplot2 package 68-70, SPSS (IBM, Armonk, NY, USA) 71. Additional exploratory analyses were conducted with Omics Playground v3.4.1^72^ and SRPlot 73-75. Quantitative data are reported as mean ± standard deviation (SD) from at least three independent experiments 73-75. Group differences were assessed using one-way or two-way analyses of variance (ANOVA), followed by Bonferroni correction for multiple comparisons 76, 77. Survival analyses were performed using the Kaplan-Meier method and compared with the log-rank test 78-80. HRs with corresponding 95% CIs were estimated using Cox proportional hazards models when appropriate. Unless otherwise indicated, statistical tests were two-sided, and a *p* value < 0.05 was considered statistically significant.

## 3. Results

### 3.1 Expression and Prognostic Relevance Extents of *AP-1* Adaptor Genes in Lung Cancer

**Figure 1:**
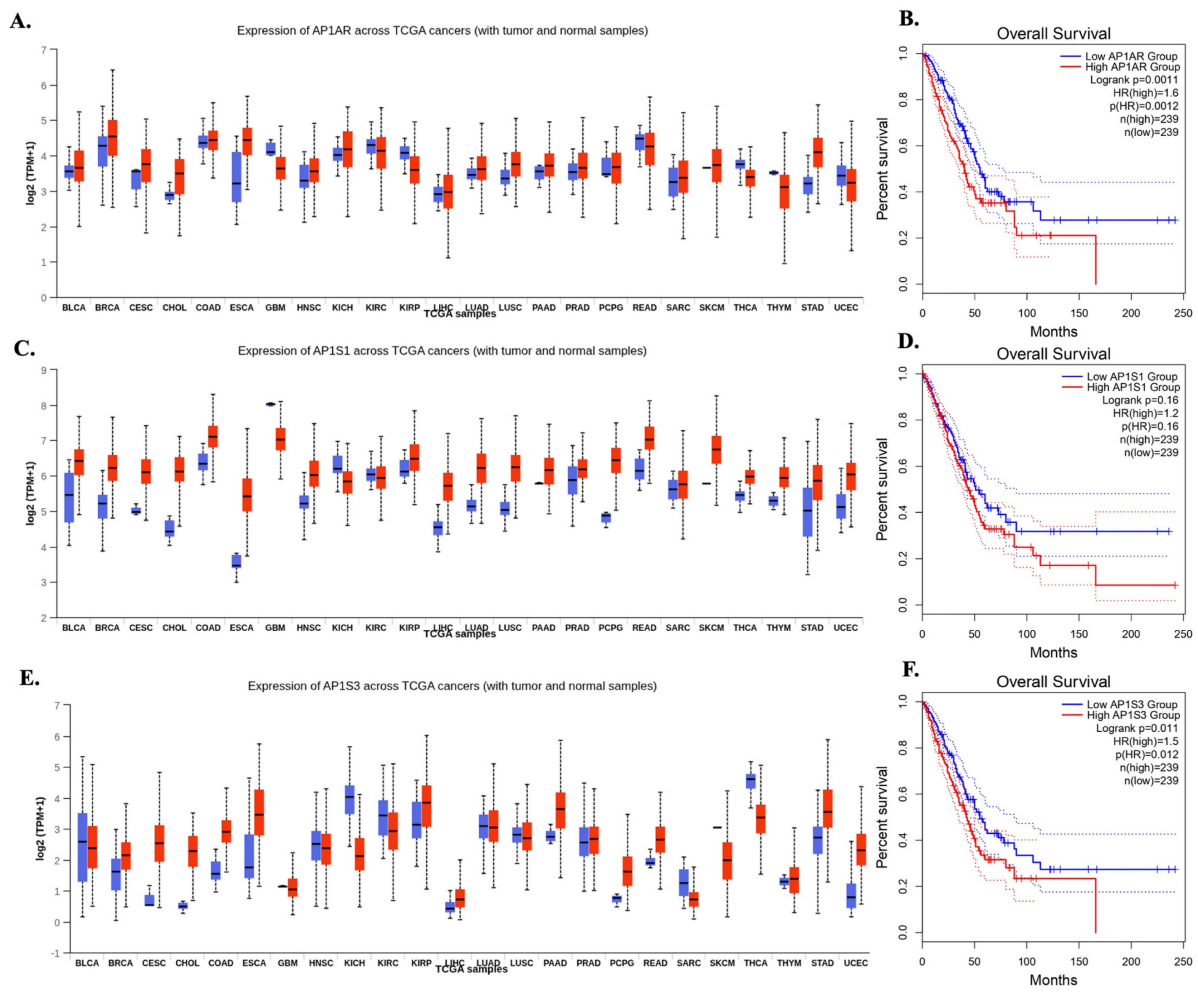
**Pan-cancer expression and overall survival associations of AP-1 adaptor genes. (A, C, E)** Boxplots showing expression of *AP1AR* (A), *AP1S1* (C), and *AP1S3* (E) across TCGA cancer types, comparing tumor (red) and normal (blue) samples. Expression is shown in log2 TPM+1. Cancer types are indicated on the x-axis. **(B, D, F)** Kaplan-Meier overall survival curves for high vs low expression of *AP1AR* (B), *AP1S1* (D), and *AP1S3* (F). Log-rank p values, Cox hazard ratios (HRs), and the number of patients in each group are indicated in the plots.

**Figure 2:**
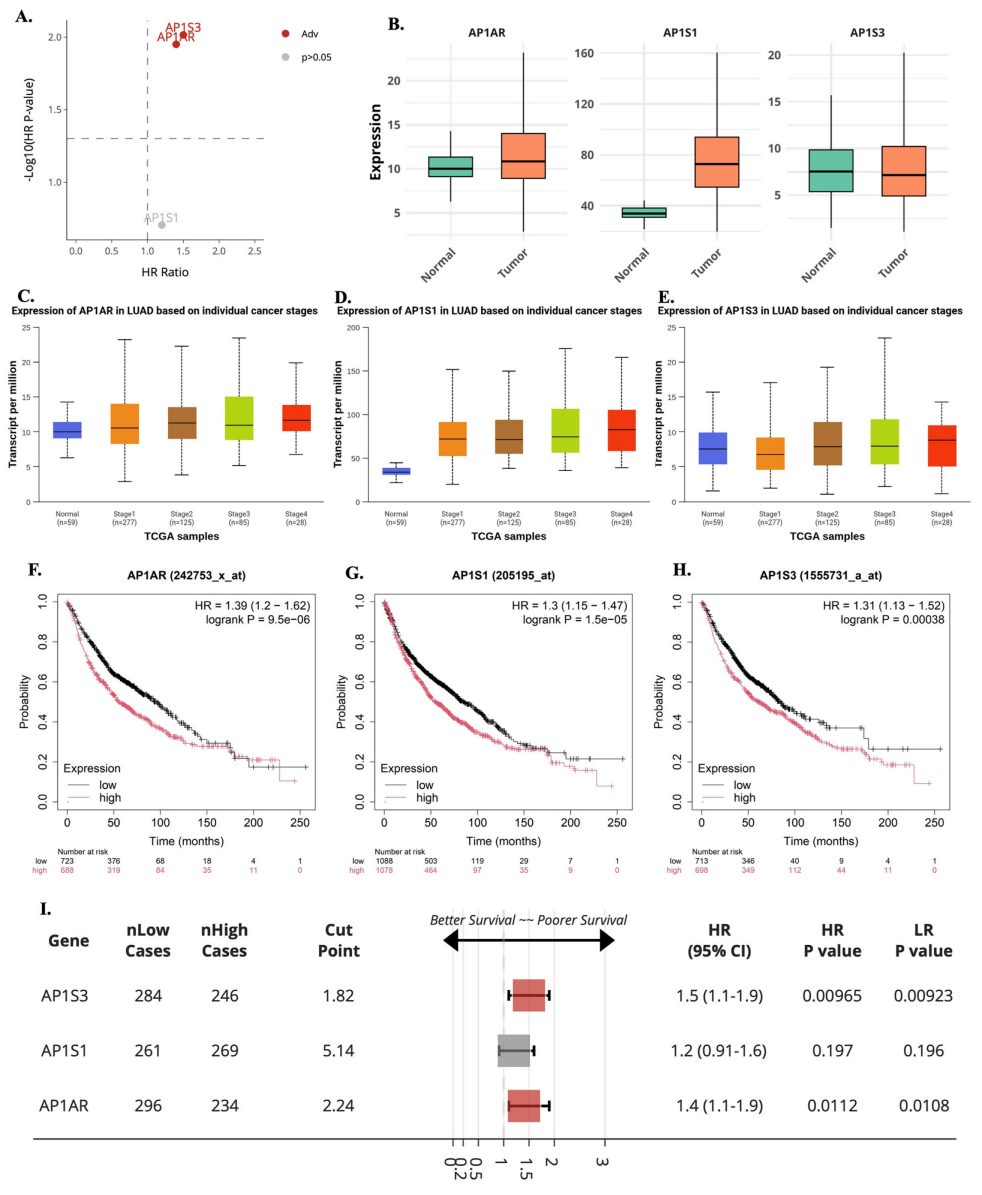
** Expression, stage distribution, and survival associations of *AP1AR, AP1S1*, and *AP1S3* in lung adenocarcinoma (LUAD). (A)** Multivariate Cox regression-based volcano plot from the SurvivalGenie platform highlighting *AP1S3* and *AP1AR* as adverse prognostic genes (HR > 1, *p* < 0.05). Gray dots indicate non-significant genes (*p* > 0.05). **(B)** Boxplots comparing *AP1AR, AP1S1*, and *AP1S3* expression between normal and LUAD tumor tissues. **(C-E)** Stage-specific expression patterns for *AP1AR* (C), *AP1S1* (D), and *AP1S3* (E), showing progressive upregulation of *AP1AR* and *AP1S3* with advancing tumor stage, while *AP1S1* remained relatively stable. **(F-H)** Kaplan-Meier overall survival curves for* AP1AR* (F), *AP1S1* (G), and *AP1S3* (H), indicating that high expression correlates with poorer survival, with *AP1AR* and *AP1S3* showing the strongest effects. **(I)** Forest plot summarizing hazard ratios (HRs) and 95% confidence intervals across datasets for *AP1S3, AP1S1* and* AP1AR*, confirming *AP1AR* and *AP1S3* as independent adverse prognostic markers in LUAD.

A systematic multi-omics analysis was conducted for the eight AP-1 adaptor complex genes (*AP1AR, AP1B1, AP1G1, AP1G2, AP1M1, AP1M2, AP1S1,* and *AP1S3*) to characterize their roles in lung cancer. These genes were investigated by integrating bulk RNA-seq, clinical outcomes, protein expression, drug-sensitivity correlations, pathway enrichment, and single-cell transcriptomics. To identify key candidates, gene expression patterns and associations with patient survival were first evaluated across TCGA datasets using the UALCAN platform. Among the eight genes, *AP1AR, AP1S1*, and *AP1S3* were consistently upregulated in LUAD relative to normal lung tissues (Figure 1A, C, E), whereas other family members showed variable or minimal changes (Supplementary Figure S1). Kaplan-Meier analyses indicated that high expression of *AP1AR* and *AP1S3* correlated with shorter overall survival (OS), with *AP1S1* showing a weaker but similar trend (Figure 1B, D, F). Multivariate Cox regression using SurvivalGenie confirmed *AP1AR* and *AP1S3* as independent adverse prognostic markers (HRs > 1, *p* < 0.05), while *AP1S1* did not reach statistical significance (Figure 2A). Pathological stage-specific analyses revealed progressive upregulation of *AP1AR* and *AP1S3* with advancing tumor stage, whereas AP1S1 expression remained relatively stable (Figure 2B-E). Kaplan-Meier survival curves further confirmed that high expression of *AP1AR*, *AP1S1*, and *AP1S3* was associated with reduced OS, with *AP1AR* and *AP1S3* showing the strongest effects (Figure 2F-H). A forest plot integrating HRs across cohorts validated *AP1AR* and *AP1S3* as independent adverse prognostic markers (Figure 2I), whereas *AP1M2* and *AP1B1* showed modest survival effects (Supplementary Figure S2). Extended analyses in TCGA-LUSC and combined TCGA-LUAD_LUSC cohorts confirmed the LUAD-specific prognostic relevance of *AP1AR* and *AP1S3* (Supplementary Figure S3A-B). DepMap gene-effect profiles indicated moderate dependency of LUAD cell lines on these genes, supporting their role in tumor viability (Supplementary Figure S3C). Integration with TCGA methylation data revealed consistent promoter hypomethylation for *AP1AR* and *AP1S3* in LUAD (Supplementary Figure S4A-C), correlating with elevated transcript levels. LUSC analyses showed weaker but directionally consistent trends (Supplementary Figure S4D-F), suggesting that transcriptional activation of these genes is at least partly epigenetically regulated.

### 3.2 Functional Pathway Enrichment of *AP1AR*, *AP1S1*, and *AP1S3*

**Figure 3:**
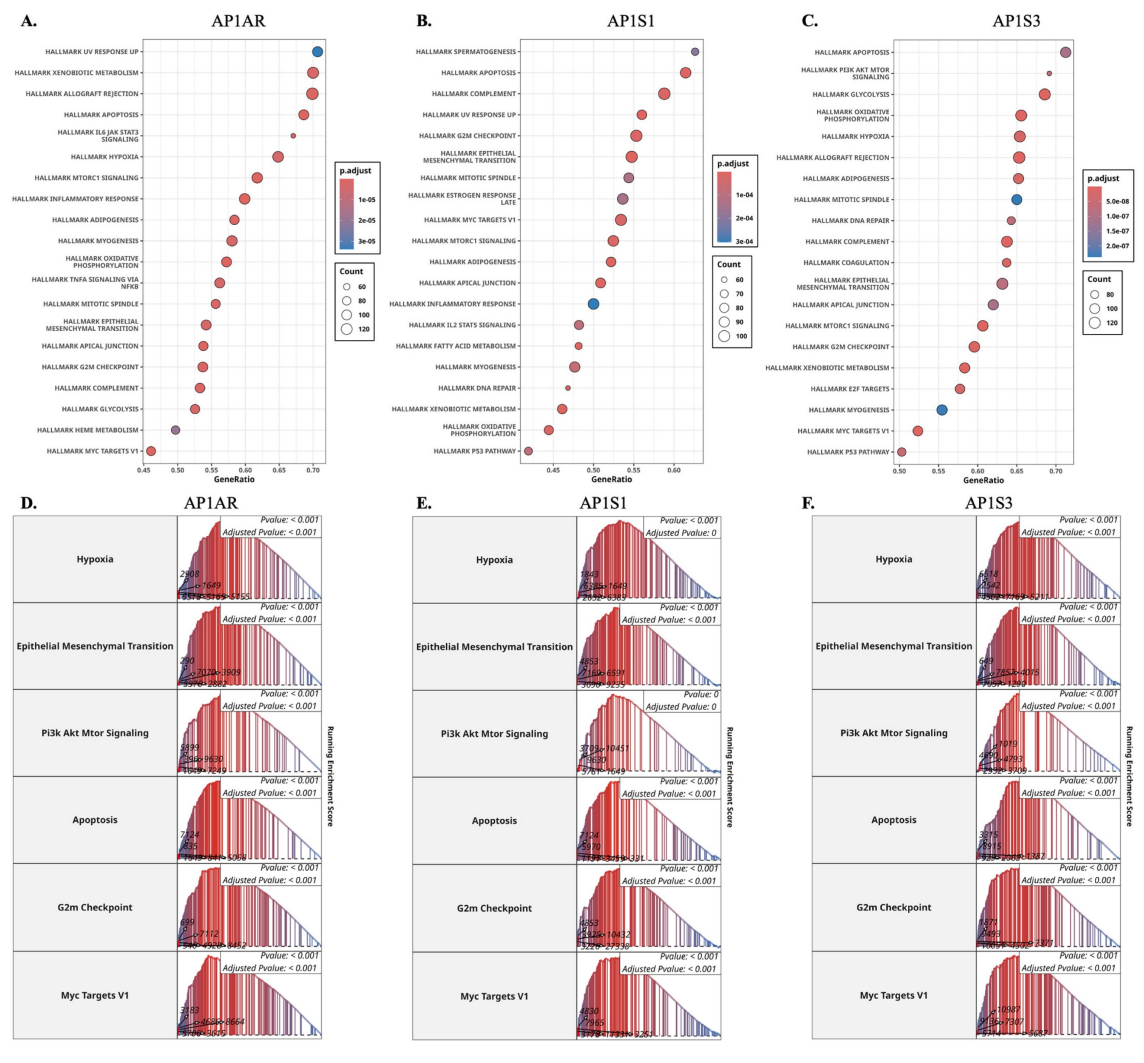
** Gene set enrichment analysis (GSEA) of AP-1 adaptor genes in lung cancer. (A-C)** Dot plots showing significantly enriched Hallmark pathways associated with *AP1AR, AP1S1*, and *AP1S3* expression in LUAD samples. Dot size represents gene count, and dot color indicates adjusted p-values. **(D-F)** Representative enrichment plots highlighting key Hallmark pathways, including Hypoxia, Epithelial-Mesenchymal Transition, PI3K/AKT/MTOR signaling, Apoptosis, G2M checkpoint, and MYC Targets V1 for *AP1AR, AP1S1*, and *AP1S3*.

To explore the biological roles of the most relevant AP-1 adaptor genes, we performed GSEA on TCGA-LUAD expression profiles. *AP1AR* was strongly enriched in pathways related to EMT, hypoxia, inflammatory response, and cell-cycle progression, highlighting its role in proliferation and tumor aggressiveness (Figure 3A, D). *AP1S1* showed enrichment for IL6-JAK-STAT3 and TNFα-NFκB signaling, as well as apoptosis and G2/M checkpoint pathways, suggesting involvement in both metabolic regulation and tumor-immune interactions (Figure 3B, E). *AP1S3* was enriched in oxidative phosphorylation, fatty acid metabolism, and cell-cycle checkpoint control, indicating a link to metabolic rewiring and stress adaptation (Figure 3C, F). Supplementary analyses confirmed that other AP-1 adaptor genes were also associated with hallmark cancer pathways, including hypoxia, apoptosis, metabolism, and EMT, supporting a broader oncogenic role for the family (Supplementary Figure S5). Parallel enrichment analyses in the TCGA-LUSC cohort showed similar patterns for *AP1AR* (Supplementary Figure S6A, D), *AP1S1* (Supplementary Figure S6B, E), and *AP1S3* (Supplementary Figure S6C, F), involving EMT, PI3K/AKT/mTOR, apoptosis, and G2/M checkpoint pathways. These results indicate that transcriptional programs associated with AP-1 adaptor dysregulation are largely conserved across lung cancer histotypes.

### 3.3 Protein-Level Validation, Drug Sensitivity Correlations, and MetaCore Analysis

**Figure 4:**
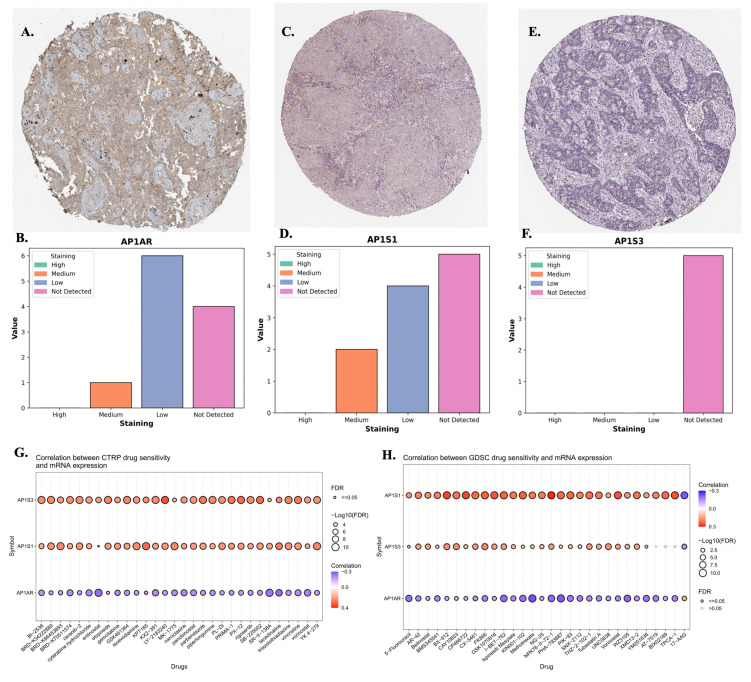
** Protein expressions and drug-sensitivity correlations of *AP1AR, AP1S1,* and *AP1S3*. (A, C, E)** Representative immunohistochemical (IHC) images from the Human Protein Atlas showing *AP1AR, AP1S1*, and *AP1S3* staining, respectively, in lung adenocarcinoma (LUAD) tissues. **(B, D, F)** Semi-quantitative distribution of staining intensities (high, medium, low, not detected) across LUAD samples, indicating that *AP1AR* is more frequently detected at the protein level than *AP1S1* and *AP1S3*. **(G)** Correlations between *AP1S3, AP1S1 and AP1AR* mRNA expression and drug sensitivity in cancer cell lines from the CTRP dataset. **(H)** Correlations between *AP1S1, AP1S3 and AP1AR* mRNA expression and drug sensitivity in the GDSC dataset. In (G-H), each bubble represents one drug; bubble color denotes the correlation coefficient (purple, negative; red, positive), and bubble size is proportional to -log10(FDR), with larger bubbles indicating stronger statistical significance.

**Figure 5:**
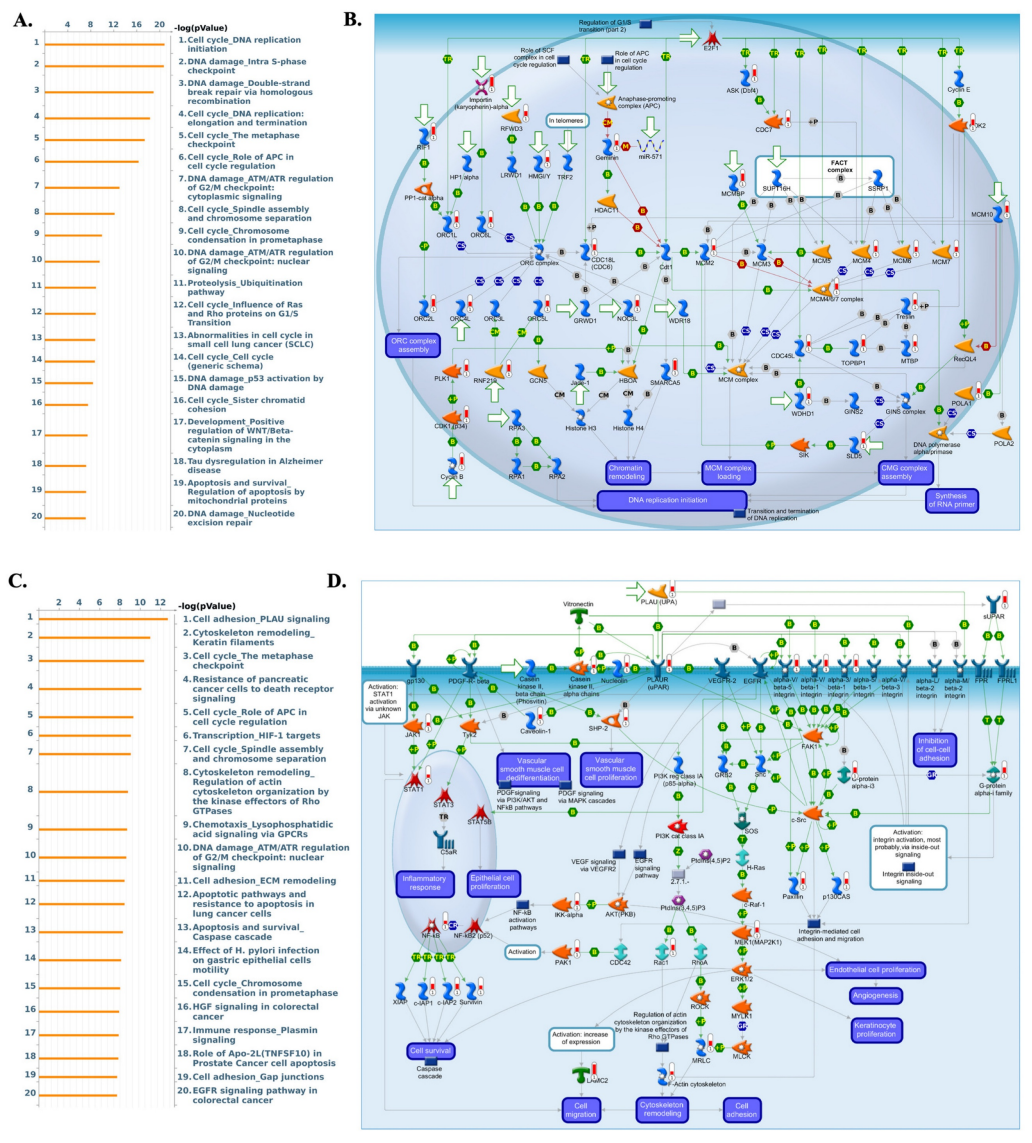
** Pathway enrichment analysis of *AP1AR*- and *AP1S3*-associated gene signatures in lung adenocarcinoma (LUAD). (A)** Bar plot of the top 20 MetaCore pathways enriched in the *AP1AR*-associated module, showing predominant enrichment of cell-cycle regulation, DNA replication, and DNA-damage checkpoint pathways (x-axis: -log10 p-value). **(B)** Representative MetaCore network illustrating *AP1AR*-related cell-cycle and DNA-replication programs, including regulation of the G1/S transition, chromatin remodeling, and MCM/CMG complex loading. **(C)** Bar plot of the top 20 MetaCore pathways enriched in the *AP1S3*-associated module, highlighting PLAU-mediated cell adhesion, cytoskeleton and ECM remodeling, apoptosis, and growth-factor signaling. **(D)** Representative MetaCore map depicting PLAU/uPAR-integrin-VEGFR/EGFR signaling and downstream cascades that regulate integrin-mediated cell adhesion, actin cytoskeleton remodeling, cell migration, and angiogenesis.

To validate transcriptomic findings, protein expression of *AP1AR, AP1S1*, and *AP1S3* was assessed using IHC data from the HPA. *AP1AR* showed detectable cytoplasmic and membranous staining in LUAD tissues, whereas *AP1S1* was weakly expressed and *AP1S3* minimally detected (Figure 4A, C, E). Quantitative summaries confirmed higher protein-level prevalence of *AP1AR* compared with the other two genes (Figure 4B, D, F). Drug sensitivity correlations were analyzed using GSCA, CTRP, and GDSC datasets. Elevated *AP1AR* expression was associated with relative resistance to multiple chemotherapeutic agents and targeted inhibitors (Figure 4G, H), whereas *AP1S1* and *AP1S3* correlated with increased sensitivity to specific small-molecule drugs, indicating differential therapeutic implications among adaptor family members. Supplementary analyses revealed that *AP1M2* and *AP1B1* were also associated with drug sensitivity in select contexts (Supplementary Figure S7). To gain mechanistic insights, MetaCore pathway analyses were performed for *AP1AR* and *AP1S3*, the two most clinically significant genes. *AP1AR* was primarily linked to cell-cycle regulation and DNA replication checkpoints, suggesting a role in sustaining tumor proliferation (Figure 5A*-*B). In contrast, *AP1S3* was associated with PI3K/AKT signaling and immune/inflammatory cross-talk pathways, highlighting its involvement in integrating oncogenic and microenvironmental signals (Figure 5C*-*D). Supplementary analyses further revealed enrichment of *AP1AR* in cytoskeletal remodeling and adhesion pathways, and *AP1S3* in extracellular matrix remodeling and adhesion pathways (Supplementary Figure S8).

### 3.4 Single-Cell Localization of AP-1 Adaptor Gene Expressions

**Figure 6:**
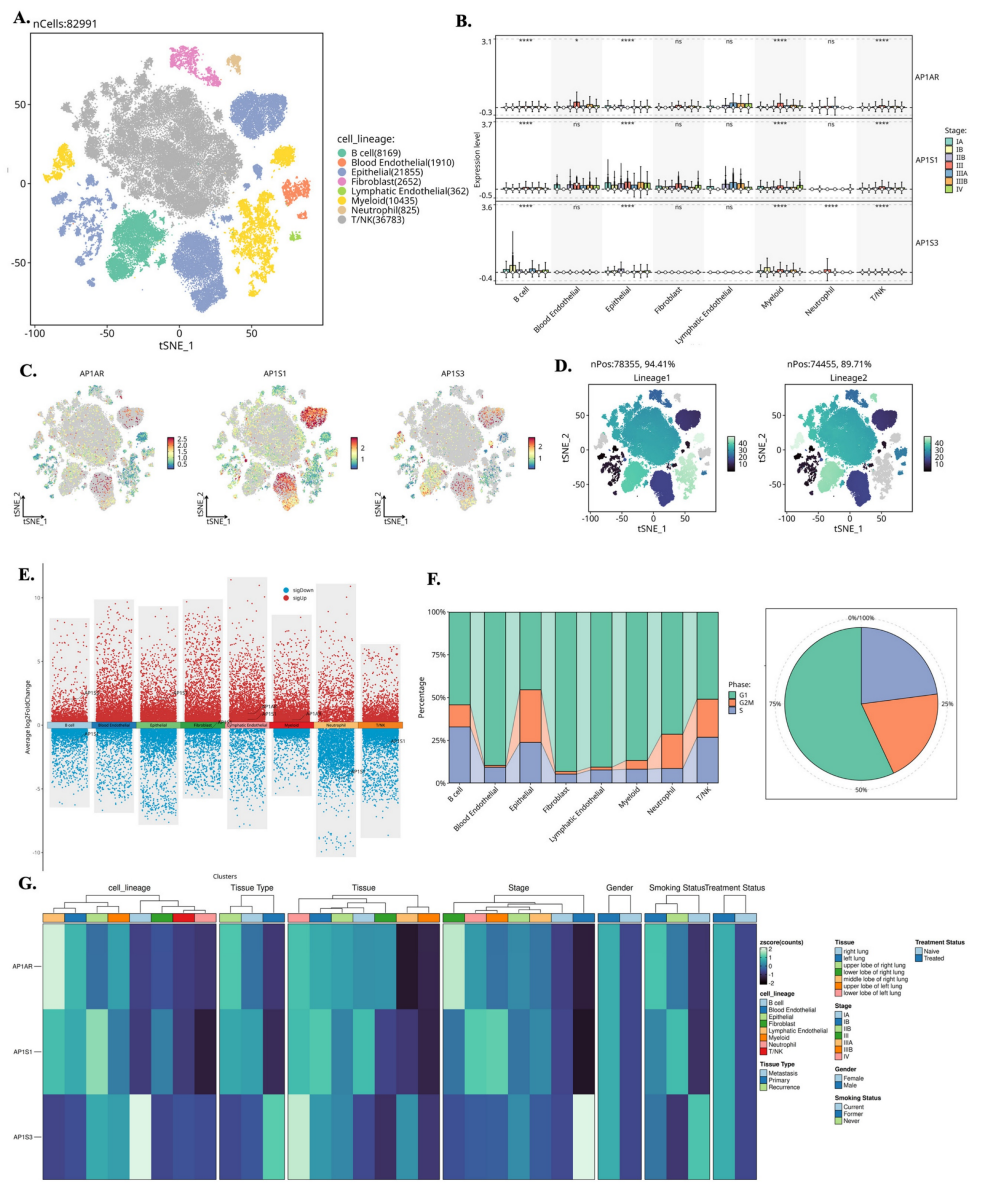
** Single-cell transcriptomic landscape of *AP1AR, AP1S1*, and *AP1S3* in lung cancer. (A)** t-SNE map of 82,991 cells from GSE202159 dataset, colored by major cell lineages. **(B)** Box plots showing normalized expression of *AP1AR, AP1S1*, and *AP1S3* across cell lineages and pathological stages, with highest expression in malignant epithelial and fibroblast cell types. **(C)** Feature plots of *AP1AR, AP1S1*, and *AP1S3* projected onto the t-SNE map, highlighting spatial localization of high-expressing clusters. **(D)** t-SNE maps illustrating lineage-level enrichment patterns (Lineage 1 and Lineage 2), indicating preferential accumulation of *AP1AR*-high cells within malignant epithelial subclusters. **(E)** Differential-expression analysis comparing *AP1AR/AP1S1/AP1S3*-high versus -low cells across lineages, shown as scatter plots of average log2 fold change; red and blue dots denote significantly upregulated and downregulated genes, respectively. **(F)** Cell-cycle phase distribution of each lineage (stacked bar plots) and of all cells combined (pie chart), indicating the proportions of cells in G1/M, G2/M, and S phases. **(G)** Heatmap of *AP1AR, AP1S1,* and *AP1S3* expression stratified by cell lineage, unsupervised cluster, tissue type (normal lung, primary tumor, recurrence), tumor stage, gender, smoking status, and treatment status, demonstrating consistent enrichment of *AP1AR* and *AP1S3* in more aggressive tumor subsets.

We analyzed single-cell RNA-seq data from GSE202159 to determine the cellular distributions and transcriptional contexts of AP-1 adaptor genes. Across ~83,000 cells, *AP1AR* and *AP1S3* were predominantly expressed in malignant epithelial and fibroblast populations, with minimal detection in immune clusters (Figure 6A-C). Lineage-specific pseudotime trajectories constructed using Slingshot revealed progressive activation of *AP1AR* along epithelial and fibroblast branches, consistent with a proliferative trajectory (Figure 6D). Differential-expression mapping highlighted widespread transcriptional upregulation associated with *AP1AR*, *AP1S1*, and *AP1S3*, implicating them in proliferative and stress-response programs (Figure 6E). Cell-cycle analysis demonstrated that most *AP1AR*- and *AP1S3*-positive cells resided in S and G2/M phases, indicating enrichment within proliferative cell types (Figure 6F). Integration with clinical annotations showed higher *AP1AR* and *AP1S3* expression in advanced-stage tumors and smoker-associated samples compared to non-tumor tissues, suggesting a link to aggressive disease phenotypes (Figure 6G). Supplementary analyses confirmed these patterns: *AP1M1, AP1B1*, and *AP1G1* exhibited moderate epithelial enrichment, whereas *AP1M2* was sparsely expressed (Supplementary Figure S9). Trajectory inferences using the SCP package revealed dynamic temporal regulation of *AP1AR* and *AP1S3.* t-SNE visualizations illustrated *AP1AR* expression across metastatic, primary, and recurrent samples, with higher signals in cells annotated as S and G2/M phases (Supplementary Figure S10A, B). Correlation analyses revealed significant associations between *AP1AR* expression and DNA-repair-related genes (Supplementary Figure S10C). Pseudotime trajectories depicted progressive *AP1AR* activation along EMT-like branches and mid-trajectory peaks for *AP1S3*, consistent with complementary roles in proliferation and stress adaptation (Supplementary Figure S10D-G).

### 3.5 Microenvironmental Communication Networks Associated with *AP1AR* Expression

**Figure 7:**
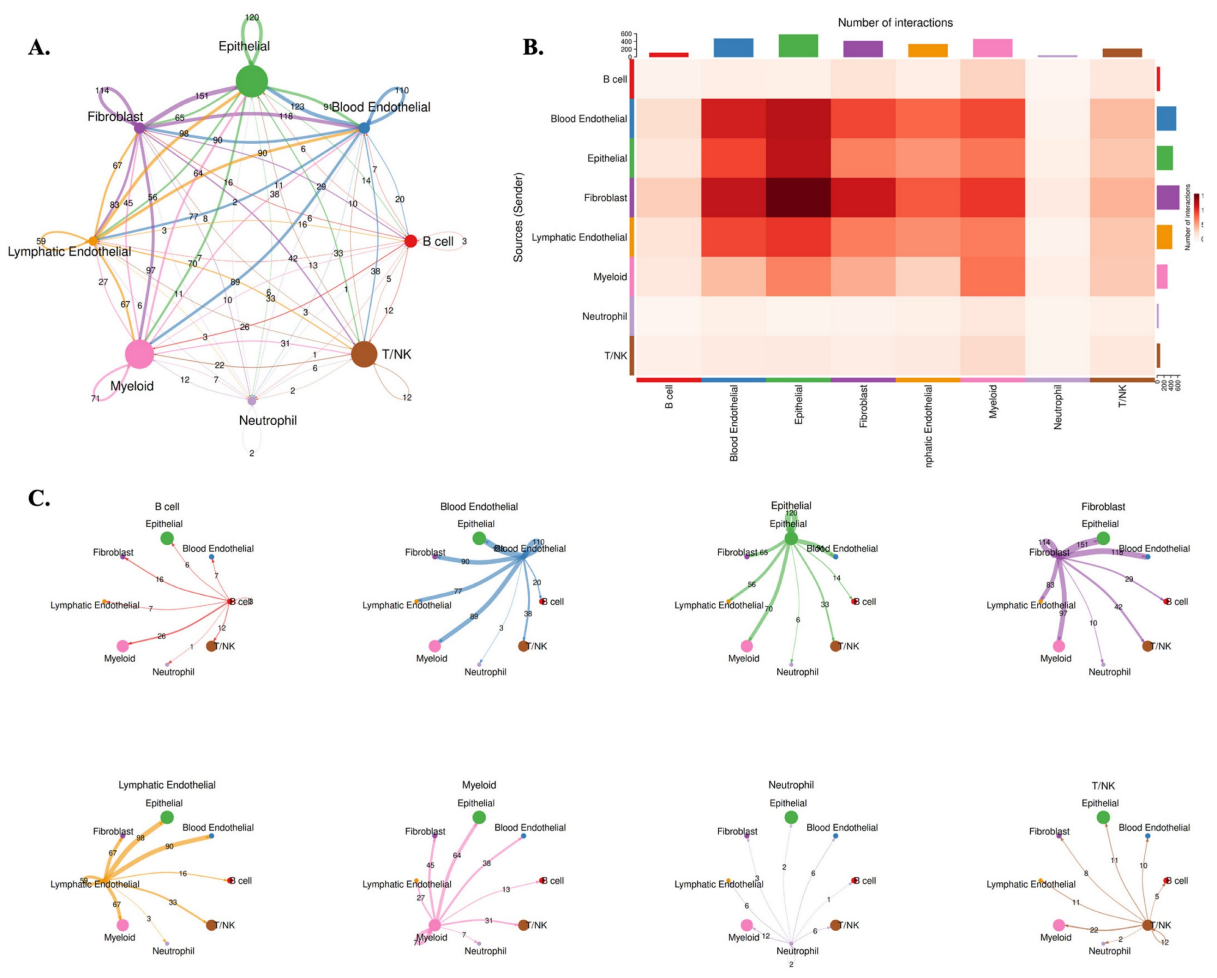
** Cell-cell communication landscape in *AP1AR*-high lung tumors. (A)** Global intercellular communication network inferred by CellChat for *AP1AR*-high samples. Nodes represent major cell lineages (B cell, blood endothelial, epithelial, fibroblast, lymphatic endothelial, myeloid, neutrophil, and T/NK), with node size proportional to the total number of incoming and outgoing interactions; edge thickness reflects the overall communication probability between lineages. **(B)** Heatmap summarizing the number of significant ligand-receptor interactions from sender (rows) to receiver (columns); darker colors indicate a higher interaction count, highlighting intensive crosstalk among epithelial, fibroblast, and endothelial cell types. **(C)** Directional network plots for each lineage, illustrating its outgoing communication to other cell types. Arrow width denotes interaction strength, revealing fibroblast-, epithelial-, and endothelial-centered signaling hubs in *AP1AR*-high lung tumors.

To investigate how *AP1AR* influences tumor-stromal interactions, we performed CellChat analysis, stratifying tumors by *AP1AR* expression. The *AP1AR*-high group exhibited markedly enhanced intercellular communication within the tumor microenvironment (TME) (Figure 7). In these tumors, fibroblast and epithelial populations acted as dominant signaling hubs, with extensive outgoing and incoming interactions involving myeloid and T/NK cells (Figure 7A-C). These interactions were enriched in growth factor- and cytokine-mediated pathways, including TGFB1-TGFBR2, IL6-IL6R, and EGF-EGFR signaling, suggesting that *AP1AR* upregulation amplifies paracrine networks supporting tumor proliferation and immune remodeling. In contrast, *AP1AR*-low tumors displayed globally reduced communication densities and weaker cross-lineage connectivity (Supplementary Figure S11), with the most pronounced loss observed in fibroblast-to-epithelial and immune-to-tumor signaling. Quantitative summaries confirmed decreased pathway activity and reduced ligand-receptor diversity, highlighting *AP1AR* expression as a key determinant of intercellular signaling intensity in lung cancer. Together, these results indicate that *AP1AR* overexpression not only drives intrinsic tumor proliferation but also promotes a communication-intensive TME, characterized by epithelial-fibroblast crosstalk and immune modulation, consistent with its enrichment in EMT and cytokine-response pathways observed in GSEA results.

## 4. Discussion

This study represents the first comprehensive analysis of the AP-1 adaptor gene family in lung cancer, integrating bulk RNA-seq, clinical outcomes, protein expressions, drug sensitivity correlations, pathway enrichment, DNA methylation, CRISPR dependency, intercellular signaling inference, and single-cell transcriptomic data. By systematically evaluating eight AP-1 adaptor genes, we identified *AP1AR* as consistently upregulated, associated with poor survival, linked to drug resistance, and enriched in hallmark oncogenic pathways. Integration of promoter methylation and CRISPR dependency profiles confirmed that this transcriptional upregulation reflects both epigenetic activation and functional relevance, underscoring the biological significance of *AP1AR*. Importantly, *AP1AR* has not previously been characterized in lung cancer, highlighting its novelty and potential clinical value.

Our analyses further validated *AP1S3* as a clinically relevant adaptor gene, linking it to oncogenic signaling and immune-related pathways, while *AP1S1* showed associations with metabolic reprogramming and immune regulation. Cross-histotype analyses in LUSC confirmed that both *AP1AR* and *AP1S3* activate convergent EMT, PI3K/AKT/mTOR, and apoptosis pathways. Single-cell transcriptomics localized *AP1AR* and *AP1S3* primarily to malignant epithelial and fibroblast cell types, particularly in proliferative states, with pseudotime trajectories revealing progressive activation along EMT-like branches. At the mechanistic level, CellChat modeling indicated that *AP1AR*-high tumors exhibit a communication-intensive tumor microenvironment, with fibroblast and epithelial populations acting as signaling hubs for immune and stromal interactions. Conversely, *AP1AR*-low tumors displayed diminished intercellular connectivity, particularly in fibroblast-to-epithelial and immune-to-tumor signaling, consistent with a dampened paracrine landscape. Functional enrichment analyses (GSEA, MetaCore) implicated *AP1AR* in cell-cycle progression, DNA replication checkpoints, hypoxia, and EMT, while *AP1S3* was linked to PI3K/AKT, KRAS, NF-κB, and MYC target pathways, connecting adaptor biology to both proliferation and immune modulation. Protein-level validation via IHC confirmed detectable *AP1AR* in tumor tissues, aligning with transcriptional and functional findings. CRISPR dependency screens indicated that *AP1AR* contributes to LUAD cell fitness, supporting a non-redundant role in tumor maintenance. Pharmacogenomic analyses revealed that *AP1AR* upregulation correlates with resistance to chemotherapeutics and targeted inhibitors, whereas *AP1S1*, *AP1S3*, *AP1M2*, and *AP1B1* showed context-dependent associations with drug sensitivity. Network analyses placed *AP1AR* centrally within AP-1 adaptor-associated protein interaction space, consistent with curated protein-protein associations and enrichment data.

Overall, these converging multi-omics findings highlight *AP1AR* and *AP1S3* as the most clinically relevant AP-1 adaptors in lung cancer, elucidating their roles in proliferation, stress adaptation, immune modulation, and drug response. This framework provides a strong foundation for biomarker development and hypothesis-driven therapy selection, offering insights into the broader contributions of AP-1 adaptor biology to lung tumorigenesis and the tumor microenvironment. Our results further reinforced these findings of *AP1S3* by linking adaptor biology to oncogenic signaling and immune pathways, while *AP1S1* showed associations with metabolic reprogramming and immune regulation. The inclusion of LUSC GSEA results strengthened this conclusion, showing that both *AP1AR* and *AP1S3* activate convergent EMT, PI3K/AKT/mTOR, and apoptosis signatures across histological subtypes. Single-cell analyses underpinned these results by grounding them in the TME, revealing that *AP1AR* and *AP1S3* are preferentially expressed in malignant epithelial and fibroblast cell types, particularly during proliferative states. An additional pseudotime reconstruction demonstrated progressive activation of *AP1AR* along EMT-like trajectories, and the CellChat analysis revealed attenuated fibroblast-epithelial and immune-tumor signaling in *AP1AR*-low contexts, suggesting that adaptor regulation may influence both proliferation and intercellular communication. These inferences are consistent with the established roles of AP-1 adaptors in TGN-to-endosome trafficking and signaling homeostasis, and with CellChat's validated framework for network-level communication inference 81.

The most interesting finding is the involvement of *AP1AR* across multiple analytic layers. *AP1AR* expression was elevated in LUAD compared to normal tissues and demonstrated a strong association with OS. We additionally observed marked promoter hypomethylation in tumor samples, supporting an epigenetic mechanism for *AP1AR* activation. Unlike other AP-1 adaptor family members, *AP1AR* has not, to our knowledge, been functionally characterized in lung cancer; nevertheless, external resources document variable tumor protein detection, which aligns with our IHC observations. CRISPR dependency screening confirmed that *AP1AR* moderately contributes to the fitness of lung-cancer cells, implying a non-redundant role in growth maintenance, and these interpretations are supported by the robustness and cross-study concordance of modern CRISPR dependency resources. GSEA and MetaCore analyses implicated *AP1AR* in cell-cycle progression, DNA-replication checkpoints, hypoxia, and EMT hallmark processes of tumor aggressiveness. Pseudotime trajectories further revealed that *AP1AR* expression peaks during G2/M and EMT-associated transitions, paralleling enrichment for DNA-repair gene signatures. Moreover, high *AP1AR* expression was correlated with resistance to chemotherapeutics and targeted agents, suggesting potential clinical implications for therapy stratification. CellChat modeling added a layer of interpretation, as *AP1AR*-low tumors exhibited globally reduced network connectivity, particularly between the stromal and epithelial cell types, consistent with a dampened paracrine signaling landscape. At the protein level, *AP1AR* was detectable by IHC, further validating its biological relevance. Single-cell analyses localized *AP1AR* to malignant epithelial clusters enriched in proliferative phases, providing strong evidence that it may act directly within tumor-driving cell types. Our analyses identified *AP1S3* as a second clinically relevant AP-1 adaptor gene in lung cancer. *AP1S3* was significantly upregulated in tumors and associated with poorer survival. Pathway analyses linked it to PI3K/AKT, KRAS, NF-κB-driven inflammatory responses, and MYC target activation, consistent with prior evidence that *AP1S3* modulates keratinocyte autophagy and enhances IL-36-dependent inflammatory signaling, connecting adaptor biology to immune pathways with potential oncogenic effects. *AP1S1* was also elevated, though with more modest survival associations; it regulates EGFR trafficking in NSCLC, and its perturbation promotes lysosomal EGFR degradation and alters TKI response, while literature links STAT3 activity to oxidative phosphorylation and therapy resistance, supporting an immune-metabolic interpretation 82, 83.

Other family members showed variable contributions. *AP1B1* and *AP1G1* participate in receptor trafficking, including EGFR polarity and recycling, and depletion of AP-1 or partners such as *GGA2* reduces EGFR surface levels and suppresses growth. AP-1 and *RAB12* cooperate in post-EGF trafficking steps that modulate downstream signaling outputs 84, 85. *AP1M2* was enriched in apoptotic and hypoxia-related pathways, whereas *AP1S2* and *AP1M1* exhibited heterogeneous expression and weaker survival associations. Pharmacogenomic analyses suggested that *AP1M2* and *AP1B1* sensitize cells to selected small-molecule inhibitors. Network analysis positioned *AP1AR* centrally within the AP-1 adaptor protein interaction network, consistent with curated protein-protein association and enrichment data. Overall, family-wide trends collectively highlight cell-cycle control, hypoxia responses, epithelial-to-mesenchymal transition, and tumor-stroma communication, providing a framework for biomarker development and hypothesis-driven therapy selection 86-88.

## 5. Conclusions

In conclusion, our integrated multi-omics and single-cell analyses identify *AP1AR* as the most consistent AP-1 adaptor signal in lung cancer, with the strongest evidence in adenocarcinoma. *AP1AR* shows transcriptional upregulation, independent associations with survival, promoter hypomethylation, enrichment of proliferation and epithelial to mesenchymal transition programs, and localization to tumor-driving cell types with altered stromal communication when low. These convergent layers nominate *AP1AR* as a clinically relevant biomarker and a candidate for translational prioritization, including hypothesis driven therapy stratification that will require prospective validation. Overall, the AP-1 adaptor complex emerges as an underexplored contributor to lung cancer biology. *AP1AR* stands out as a tractable focus for future mechanistic studies, biomarker development, and clinical evaluation aimed at therapy selection guided by adaptor gene expression.

## Supplementary Material

Supplementary figures.
