# Supplementary material for: Comprehensive characterization of AP-1 adaptor complex genes in lung cancer reveals AP1AR as a novel prognostic and therapeutic biomarker
Source: J Cancer. 2026 Jan 1;17(1):142–56. doi: 10.7150/jca.125763 (PMC12719575; doi:10.7150/jca.125763)
Supplement: Supplementary file 1 — Supplementary figures. [file jcav17p0142s1.pdf]

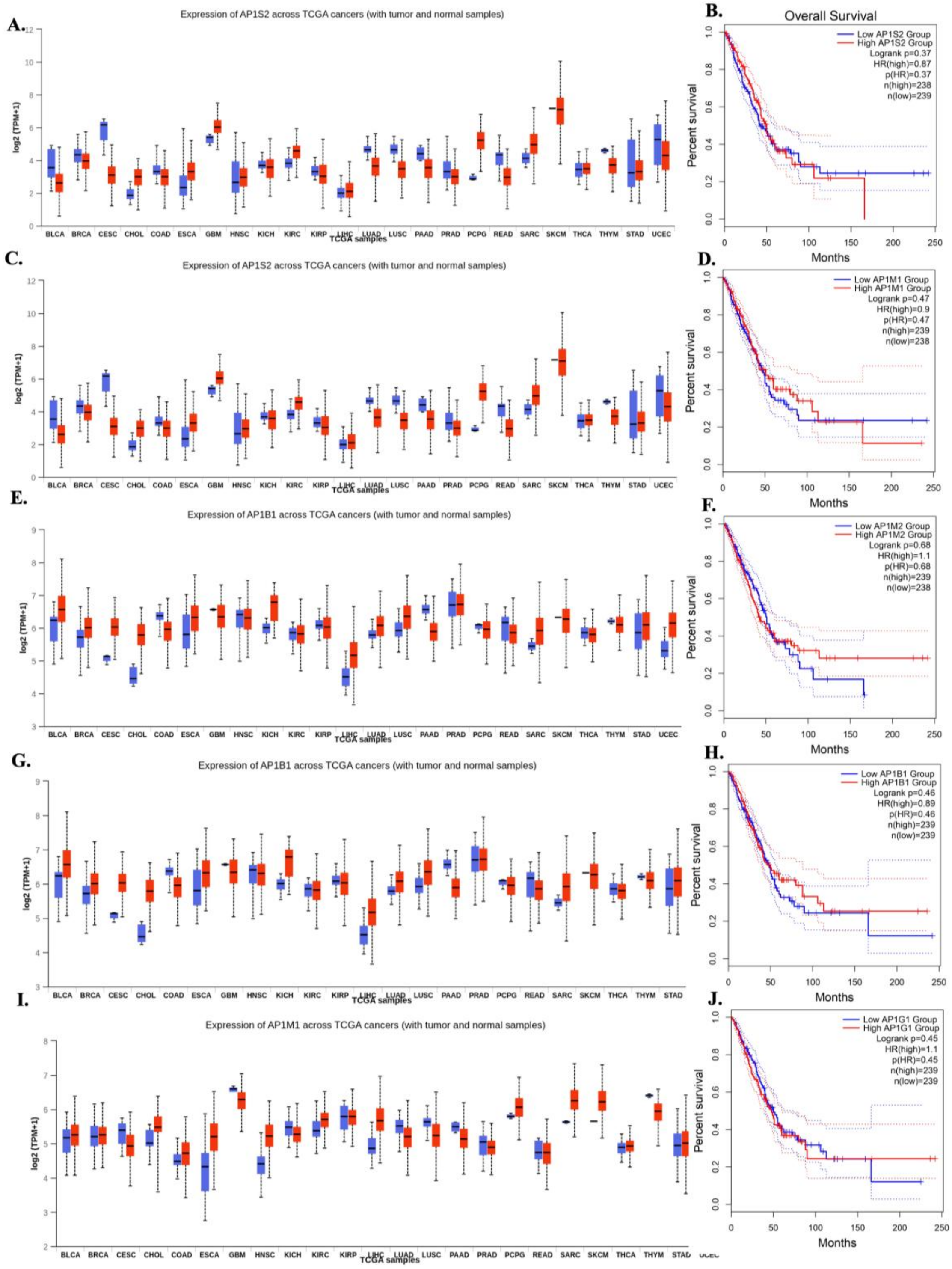

Supplementary Figure S1: Extended survey of additional AP-1 adaptor complex genes across human cancers. (A, C, E, G, I) Pan-cancer expression profiles of *AP1S2, AP1M1, AP1M2, AP1B1*, and *AP1G1*, respectively, using TCGA RNA-seq data. Boxplots display log2(TPM+1) expression values in tumor (red) and matched normal (blue) tissues across multiple cancer types. **(B, D, F, H, J)** Kaplan–Meier overall-survival curves for TCGA-LUAD patients stratified into high- and low-expression groups (median cutoff) for *AP1S2, AP1M1, AP1M2, AP1B1*, and *AP1G1*, respectively. Solid lines indicate survival probabilities over time (months), with dotted lines showing 95% confidence intervals. Log-rank p values, hazard ratios, and group sizes (n) are shown within each panel, illustrating the prognostic impact of each AP-1 adaptor gene in lung adenocarcinoma.

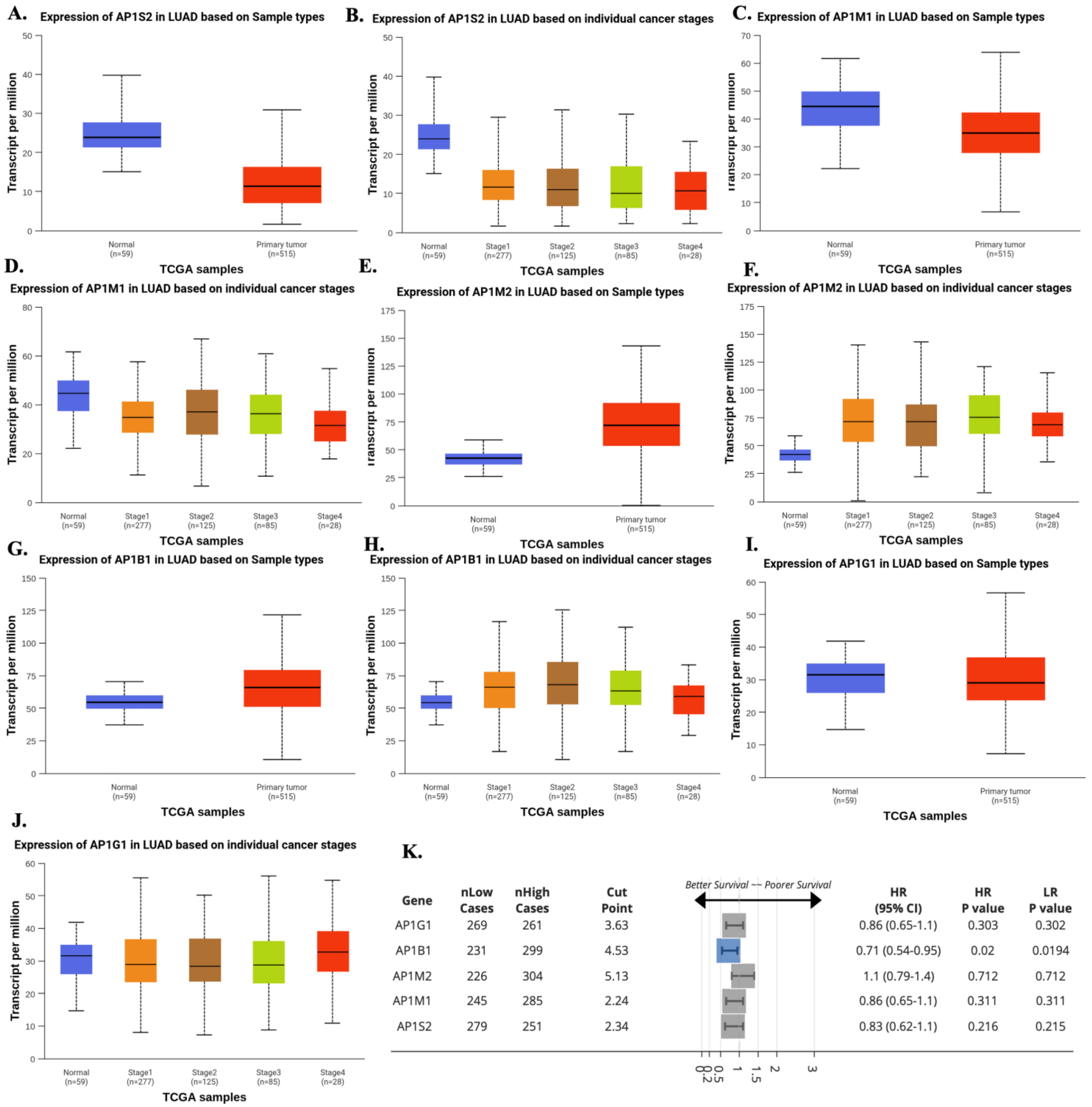

Supplementary Figure S2: Extended expression and survival analyses of additional AP-1 adaptor genes in lung adenocarcinoma (LUAD). **(A, B)** Boxplots showing *AP1S2* expression in TCGA-LUAD: normal versus primary tumors (A) and across pathological stages I–IV (B), expressed as transcripts per million (TPM). **(C, D)** *AP1M1* expression in normal versus primary LUAD samples (C) and by cancer stage (D). **(E, F)** *AP1M2* expression in normal versus primary tumors (E) and across LUAD stages (F). **(G, H)** *AP1B1* expression in normal versus primary tumors (G) and by stage (H). **(I, J)** *AP1G1* expression in normal versus primary tumors (I) and by stage (J). **(K)** Summary table and forest plot of overall-survival analyses for *AP1G1, AP1B1, AP1M2, AP1M1*, and *AP1S2* in TCGA-LUAD. The table lists the numbers of patients in low- and high-expression groups and the optimal expression cut-points (TPM). The forest plot displays hazard ratios (HRs) with 95% confidence intervals for high versus low expression, with the horizontal axis indicating better to poorer survival. *AP1B1* high

expression is associated with improved survival (HR = 0.71, 95% CI 0.54-0.95; log-rank P = 0.02), whereas the other genes show no statistically significant association with overall survival.

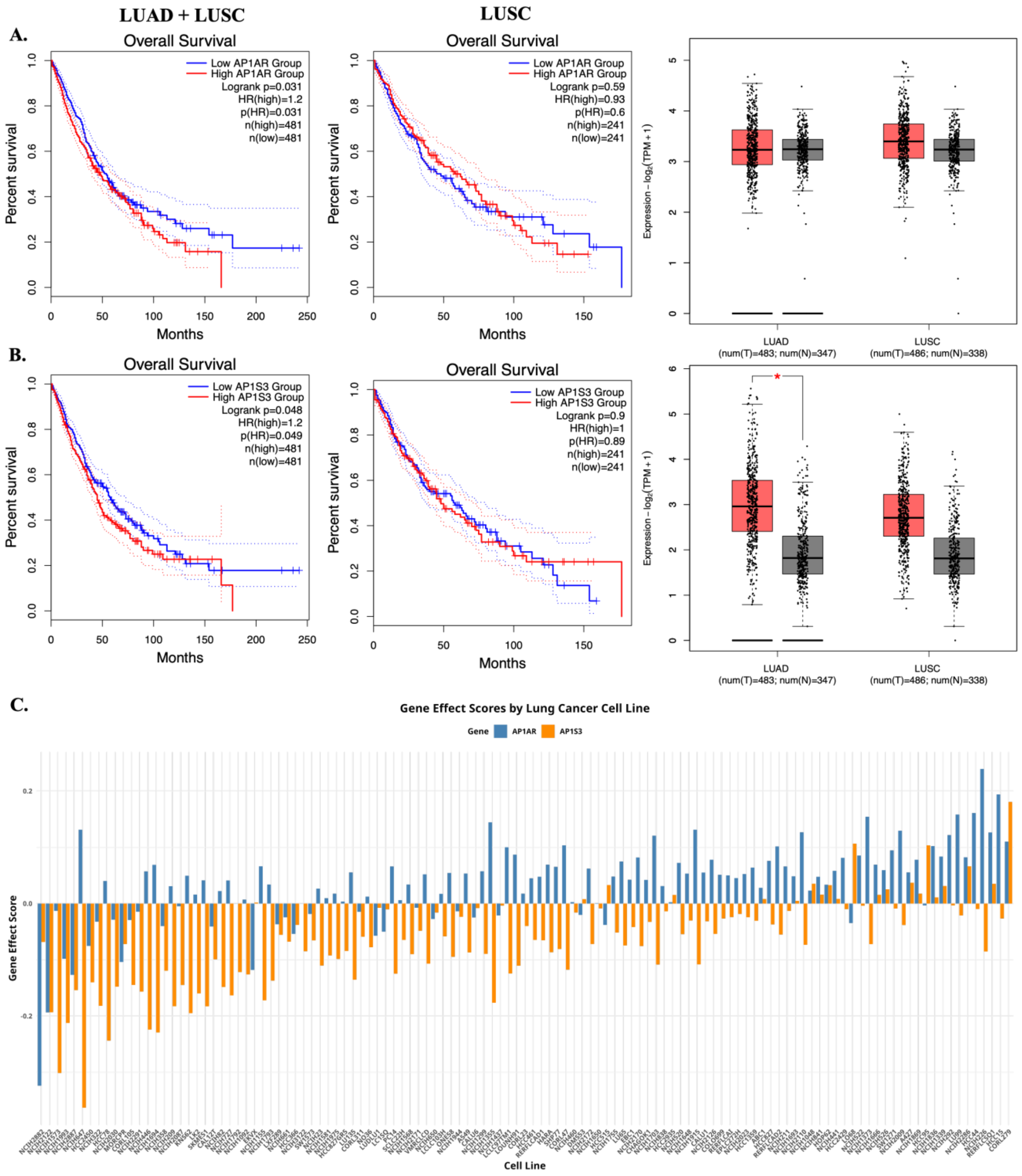

Supplementary Figure S3: Extended survival and dependency analyses of *AP1AR* and *AP1S3* across lung adenocarcinoma (LUAD) and lung squamous cell carcinoma (LUSC) cohorts. (A) Kaplan–Meier survival curves showing that high AP1AR expression predicts poor overall survival in combined LUAD and LUCS but not in LUSC. **(B)** Equivalent survival curves for AP1S3 highlighting a LUAD-dominant prognostic effect. **(C)** CRISPR–Cas9 gene-dependency (CERES) scores for *AP1AR* (blue) and *AP1S3* (orange) across a panel of LUAD and LUSC cell lines from DepMap. Negative scores reflect stronger dependency, indicating that many lung cancer cell lines are more reliant on *AP1AR* than on *AP1S3* for proliferation or survival**.**

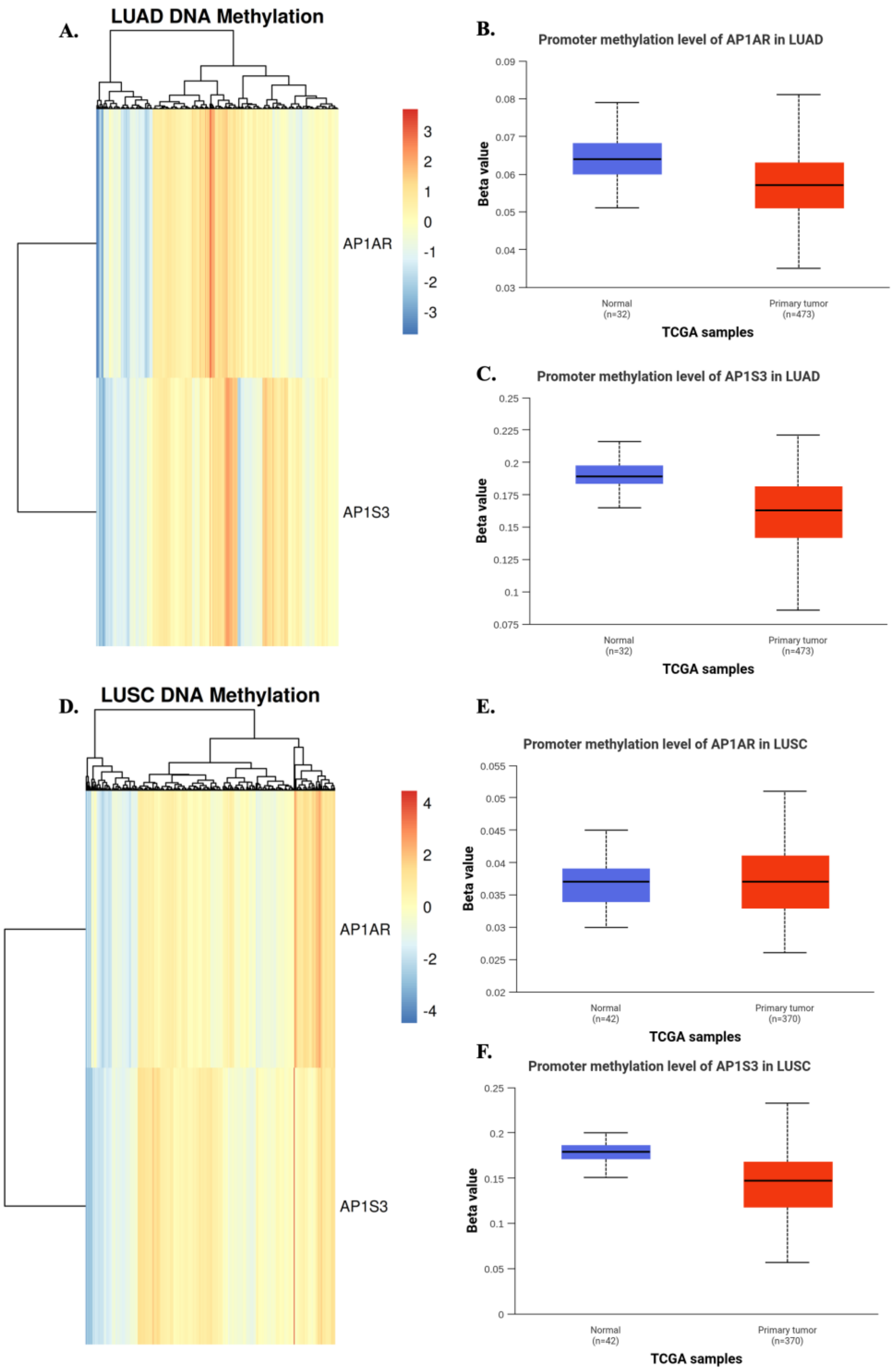

Supplementary Figure S4: DNA methylation profiles of *AP1AR* and *AP1S3* in TCGA lung adenocarcinoma (LUAD) and lung squamous cell carcinoma (LUSC). (A) Hierarchical-clustering heatmap of CpG methylation β-values for *AP1AR* and *AP1S3* in TCGA-LUAD. Columns represent individual samples and rows represent genes; colors indicate z-scored β-values (blue, hypomethylated; red, hypermethylated). **(B, C)** Boxplots comparing promoter methylation levels of *AP1AR* (B) and *AP1S3* (C) between normal lung tissues (blue, n = 32) and primary LUAD tumors (red, n = 477). **(D)** Hierarchical-clustering heatmap of CpG methylation β-values for *AP1AR* and *AP1S3* in TCGA-LUSC, shown as in (A). **(E, F)** Boxplots comparing promoter methylation levels of *AP1AR* (E) and *AP1S3* (F) between normal lung tissues (blue, n = 42) and primary LUSC tumors (red, n = 370).

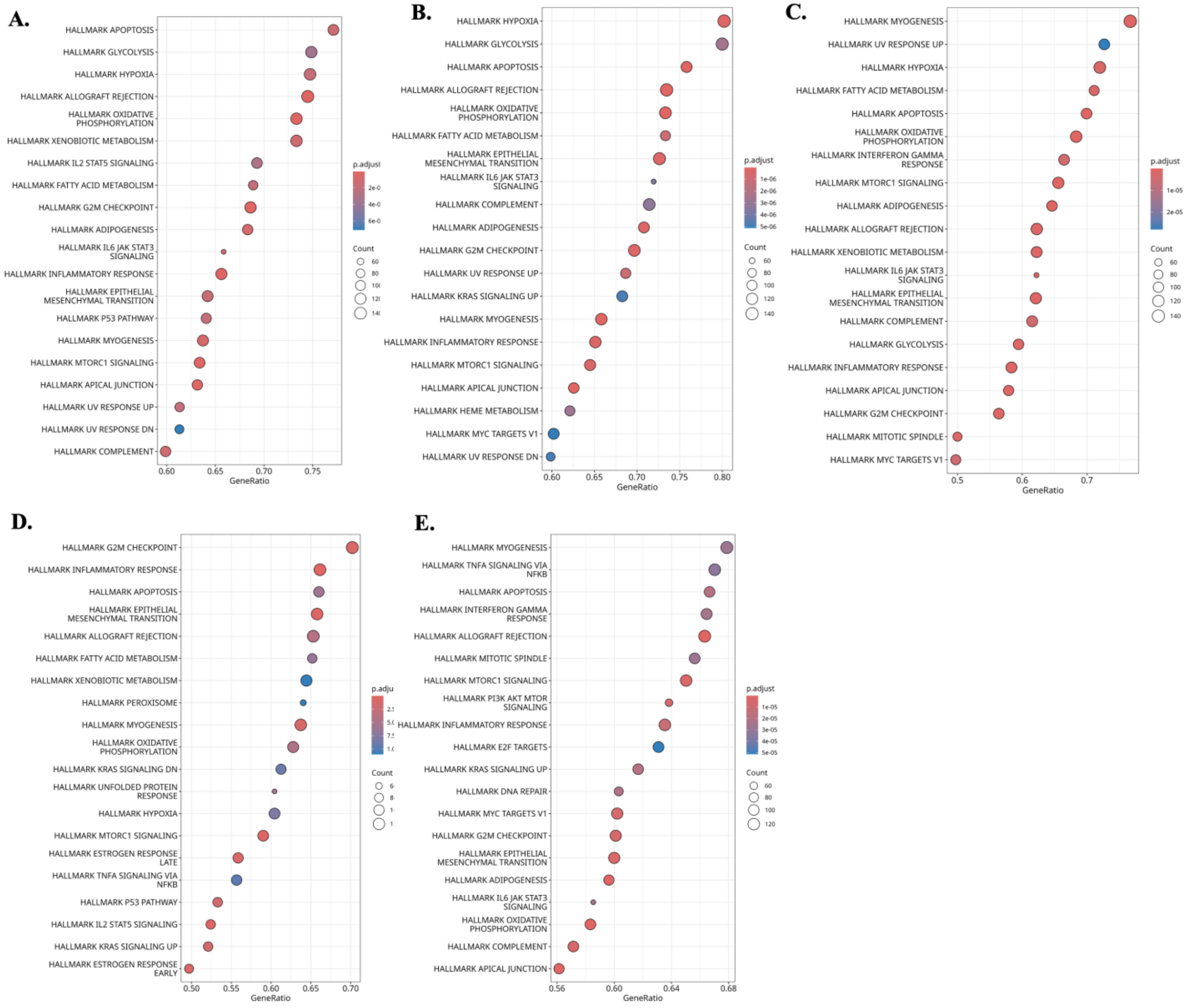

Supplementary Figure S5: Extended gene set enrichment analysis (GSEA) of additional *AP-1* adaptor genes. (A–E) Dot plots showing significantly enriched Hallmark pathways associated with AP1AR, AP1S1, and AP1S3 expression in LUAD samples. Dot size represents gene count, and dot color indicates adjusted p-values.

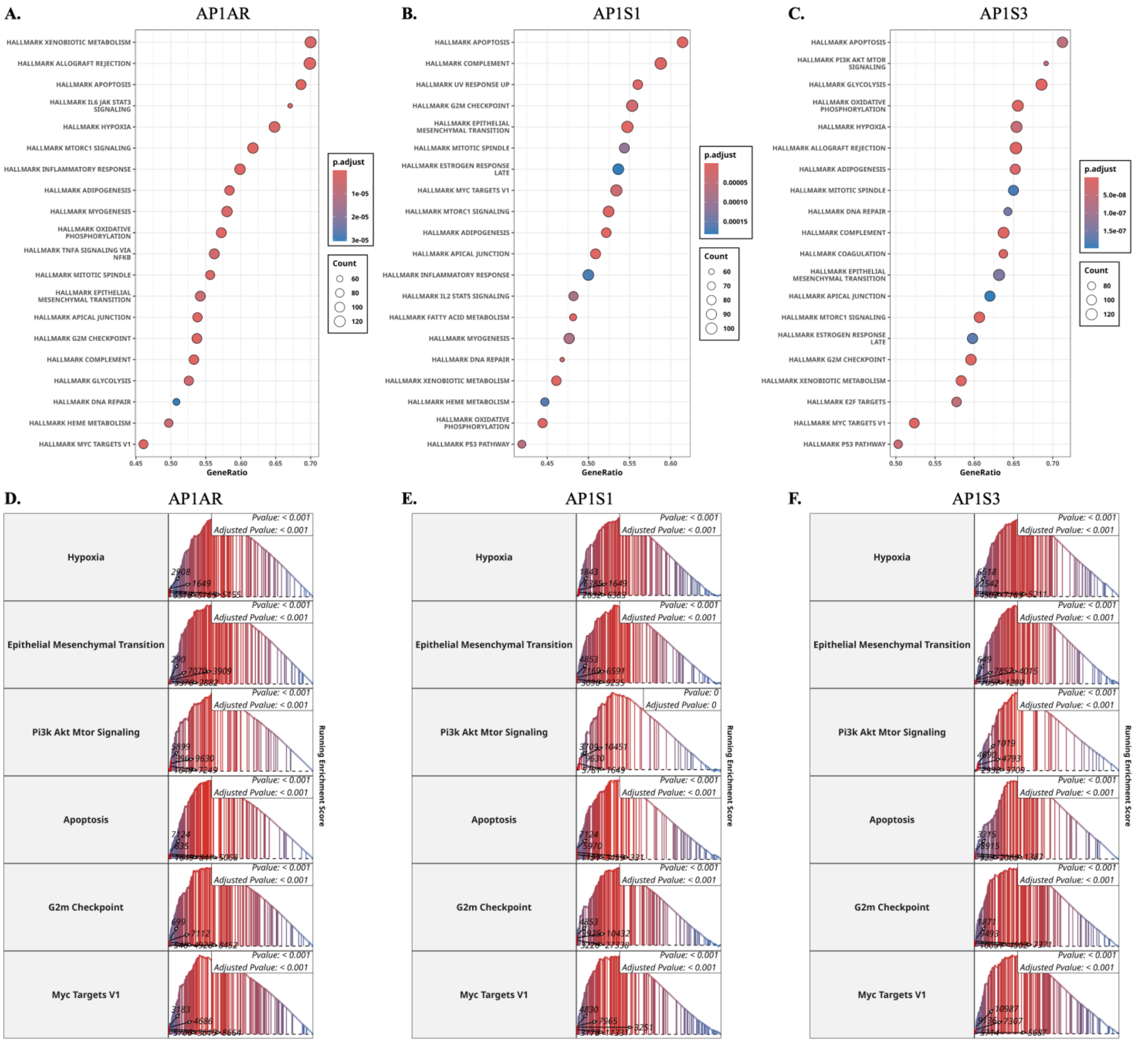

Supplementary Figure S6: Gene set enrichment analyses (GSEAs) of *AP1AR*, *AP1S1*, and *AP1S3* in TCGA-LUSC. (A–C) Dot plots showing significantly enriched Hallmark pathways associated with AP1AR, AP1S1, and AP1S3 expression in LUAD samples. Dot size represents gene count, and dot color indicates adjusted p-values. **(D–F)** Representative enrichment plots highlighting key Hallmark pathways, including Hypoxia, Epithelial–Mesenchymal Transition, PI3K/AKT/MTOR signaling, Apoptosis, G2M checkpoint, and MYC Targets V1 for AP1AR, AP1S1, and AP1S3.

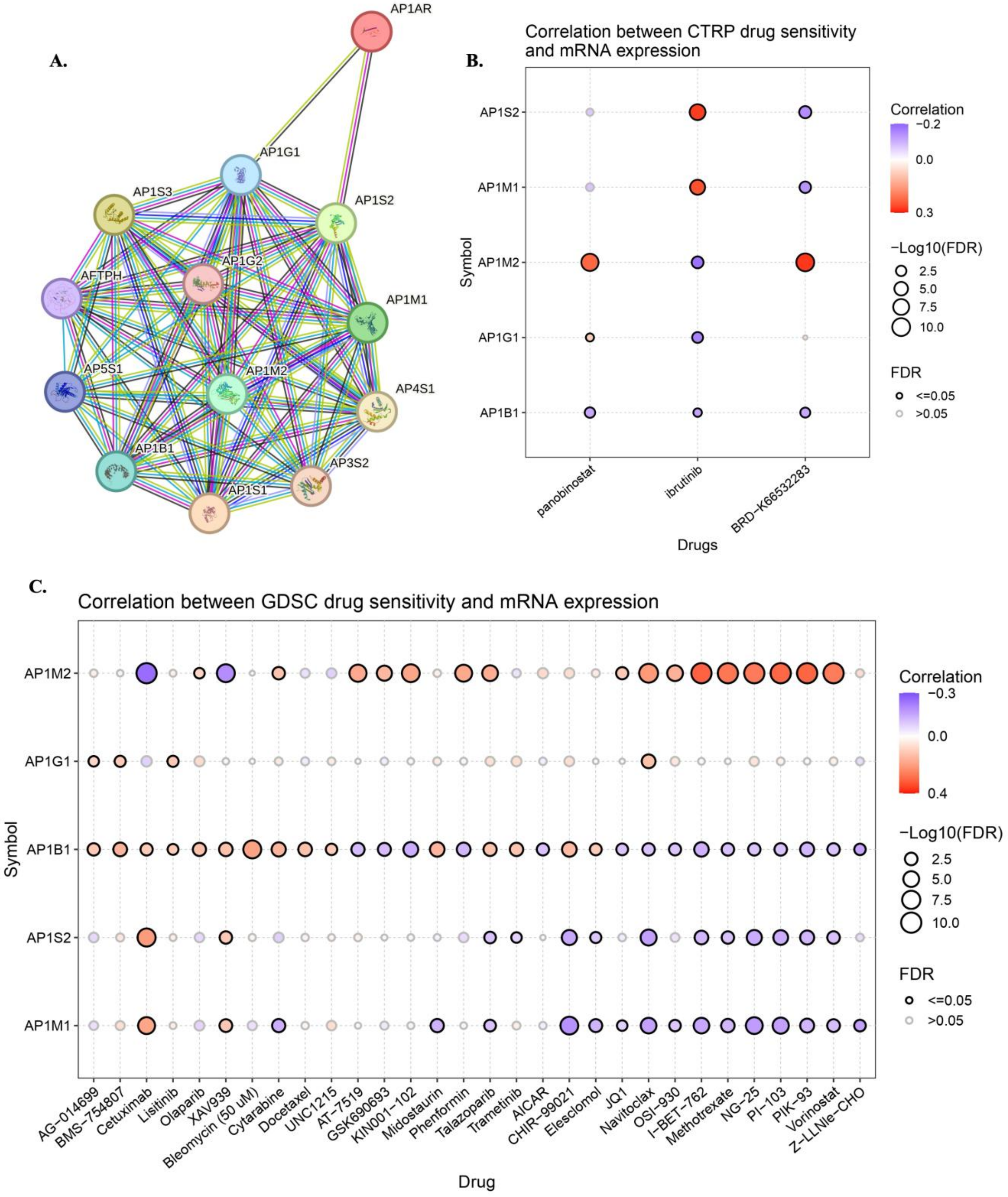

Supplementary Figure S7: Interaction network and extended drug-sensitivity analysis of additional AP-1 adaptor genes. **(A)** Protein–protein interaction network of AP-1 adaptor complex members generated using STRING. Nodes represent individual AP-1 subunits, with edge thickness reflecting the confidence of functional associations. **(B)** Bubble plot shows correlations between mRNA expression of *AP1S2, AP1M1, AP1M2, AP1G1*, and *AP1B1* and drug sensitivity in cancer cell lines from the CTRP dataset. **(C)** Correlations between mRNA expression of *AP1S2, AP1M1, AP1M2, AP1G1*, and *AP1B1* sensitivity in the GDSC dataset. Each bubble represents one gene-drug pair; bubble color indicates the Pearson correlation coefficient (blue, negative; red, positive), and bubble size is proportional to −log10(FDR), with larger bubbles denoting stronger statistical significance.

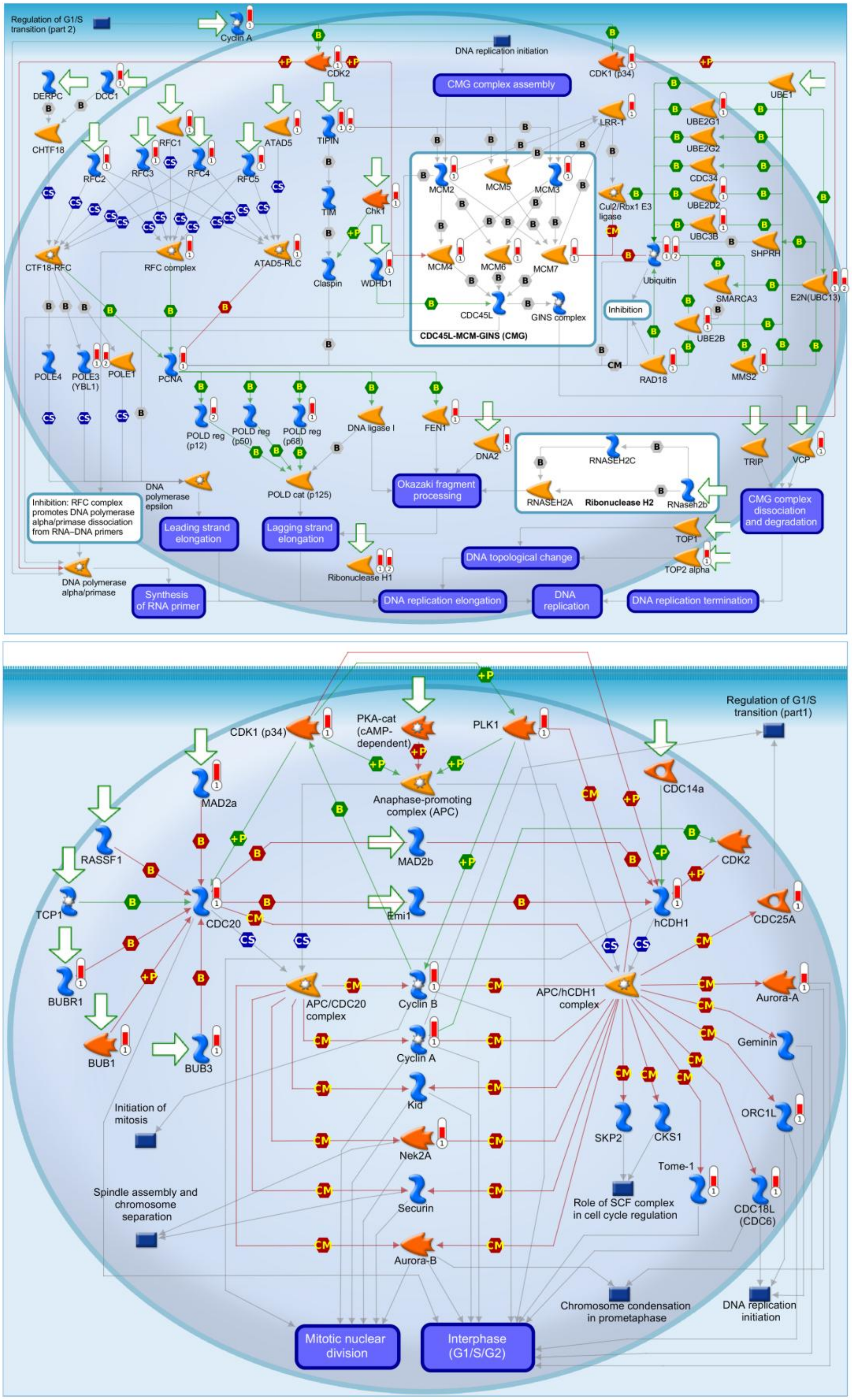

Supplementary Figure S8: Additional pathways enriched for *AP1AR* and *AP1S3*. (A) *AP1AR* showed enrichment in cytoskeletal remodeling and adhesion-related pathways, suggesting roles in invasion and metastatic spread. **(B)** *AP1S3* exhibits enrichment in cell adhesion and extracellular matrix (ECM) remodeling pathways, underscoring its role in tumor-stromal interactions.

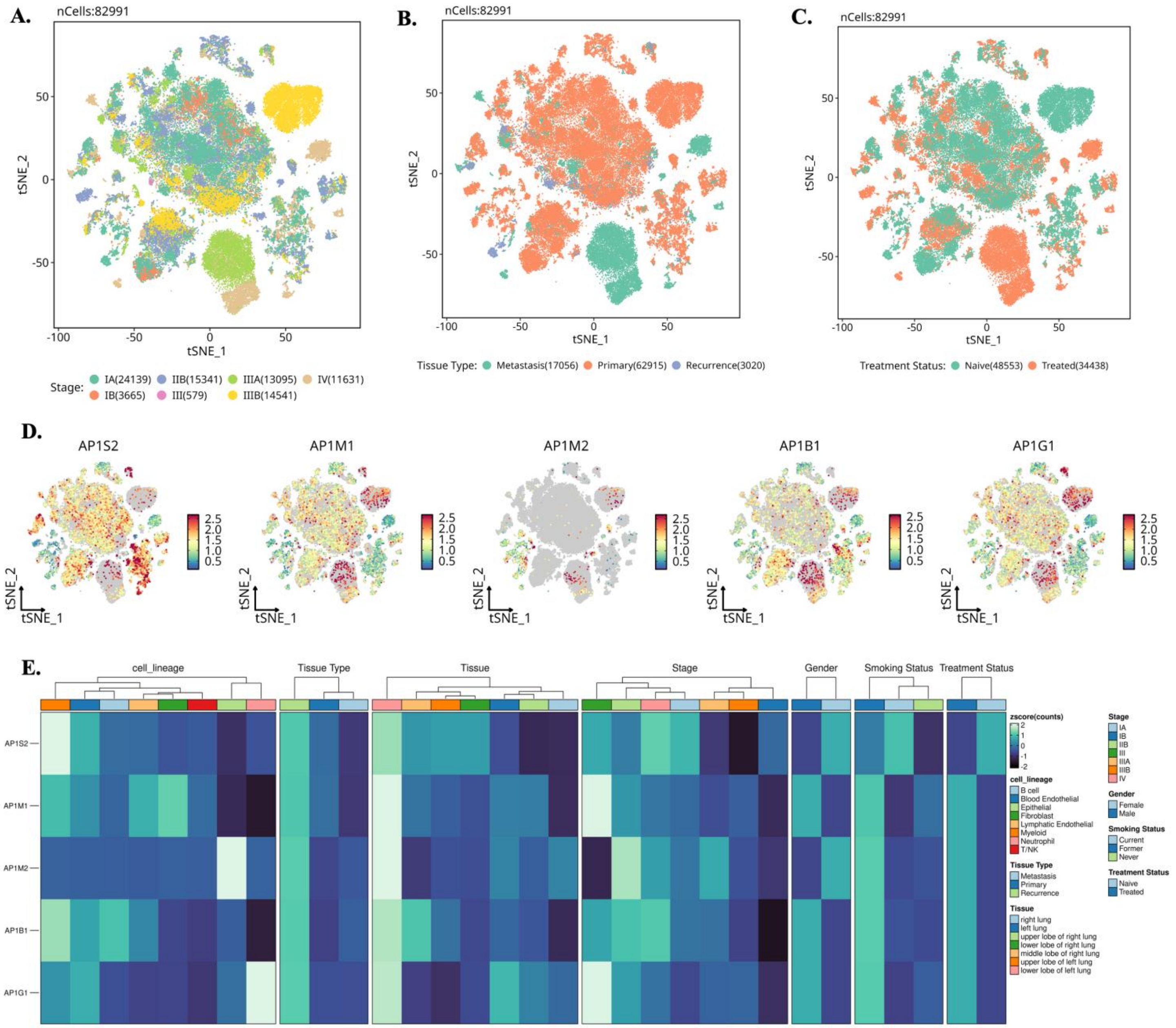

Supplementary Figure S9: Extended single-cell characterization of additional AP-1 adaptor genes in lung cancer. (A–C) t-SNE plots showing sample distributions by tumor stage, tissue type (primary, metastasis, and recurrence), and treatment status. **(D)** Feature plots of *AP1S2, AP1M1, AP1M2, AP1B1,* and *AP1G1*, demonstrating heterogeneous expressions across epithelial and immune cell types. **(E)** Heatmap of supplementary AP-1 genes across clinical and cellular covariates (lineage, tissue type, stage, smoking, and treatment), showing that *AP1M1, AP1B1,* and *AP1G1* displayed moderate but consistent enrichment in malignant epithelial cells, while *AP1M2* expression was sparser.

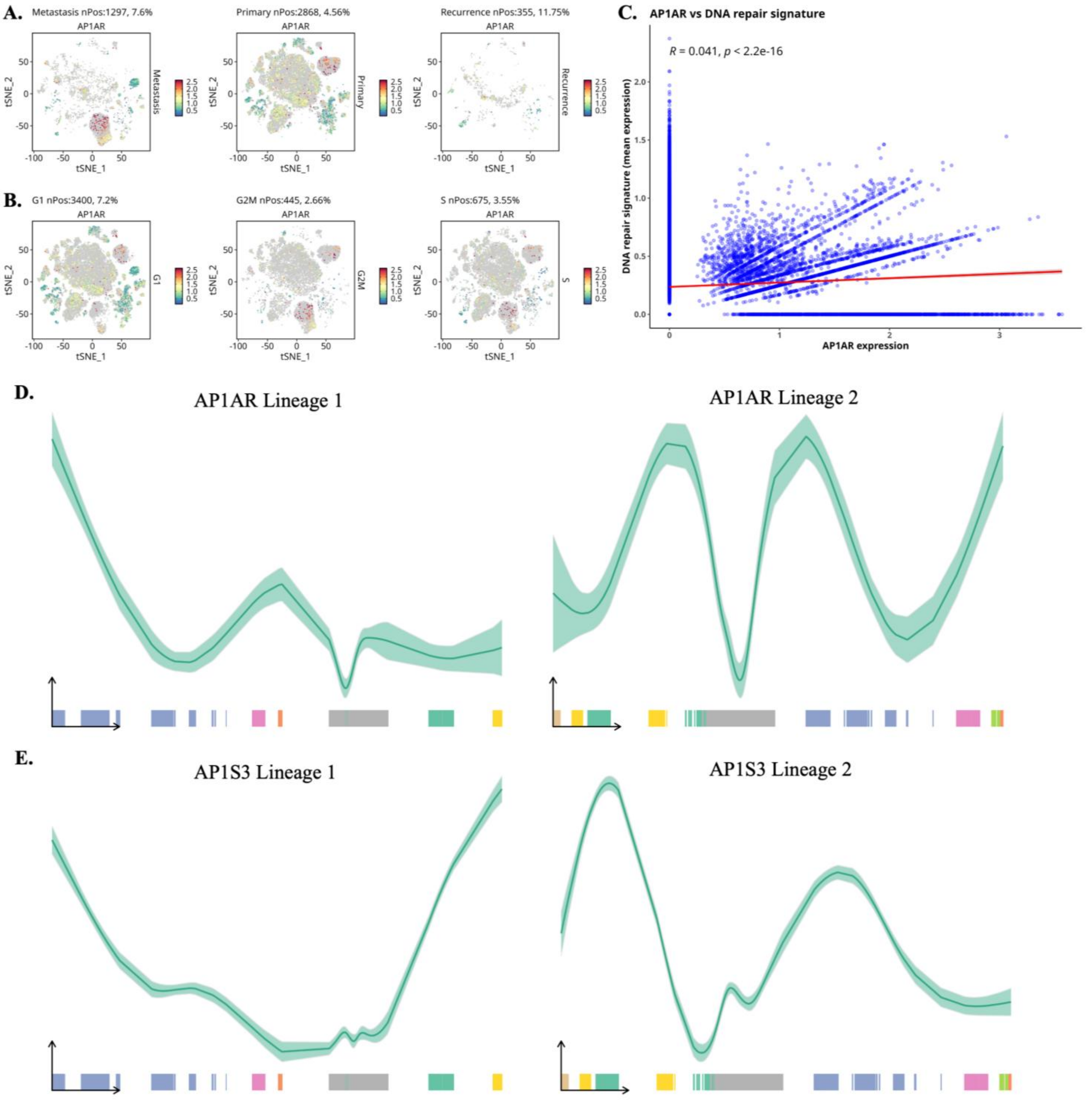

Supplementary Figure S10: Pseudotime and lineage trajectory analysis of *AP1AR* and *AP1S3* using Slingshot (in the SCP package). (A) t-SNE visualizations showing *AP1AR* expression across metastatic, primary, and recurrent samples. **(B)** Cell-cycle stratified t-SNE overlays (G1, G2/M, and S phases). **(C)** Correlation between *AP1AR* expression and a DNA-repair gene signature. *AP1AR* exhibited a positive association ($R = 0.041$, $p < 2.2 \times 10^{-16}$), indicating a connection between adaptor dysregulation and replication-stress response programs. **(D)** Slingshot lineage trajectory (lineage 1) showing smoothed pseudotime expression curves for *AP1AR*. **(E)** Slingshot lineage trajectory (lineage 2) for *AP1AR*. **(F)** Slingshot lineage 1 trajectory for *AP1S3*. **(G)** Slingshot lineage 2 trajectory for *AP1S3*.

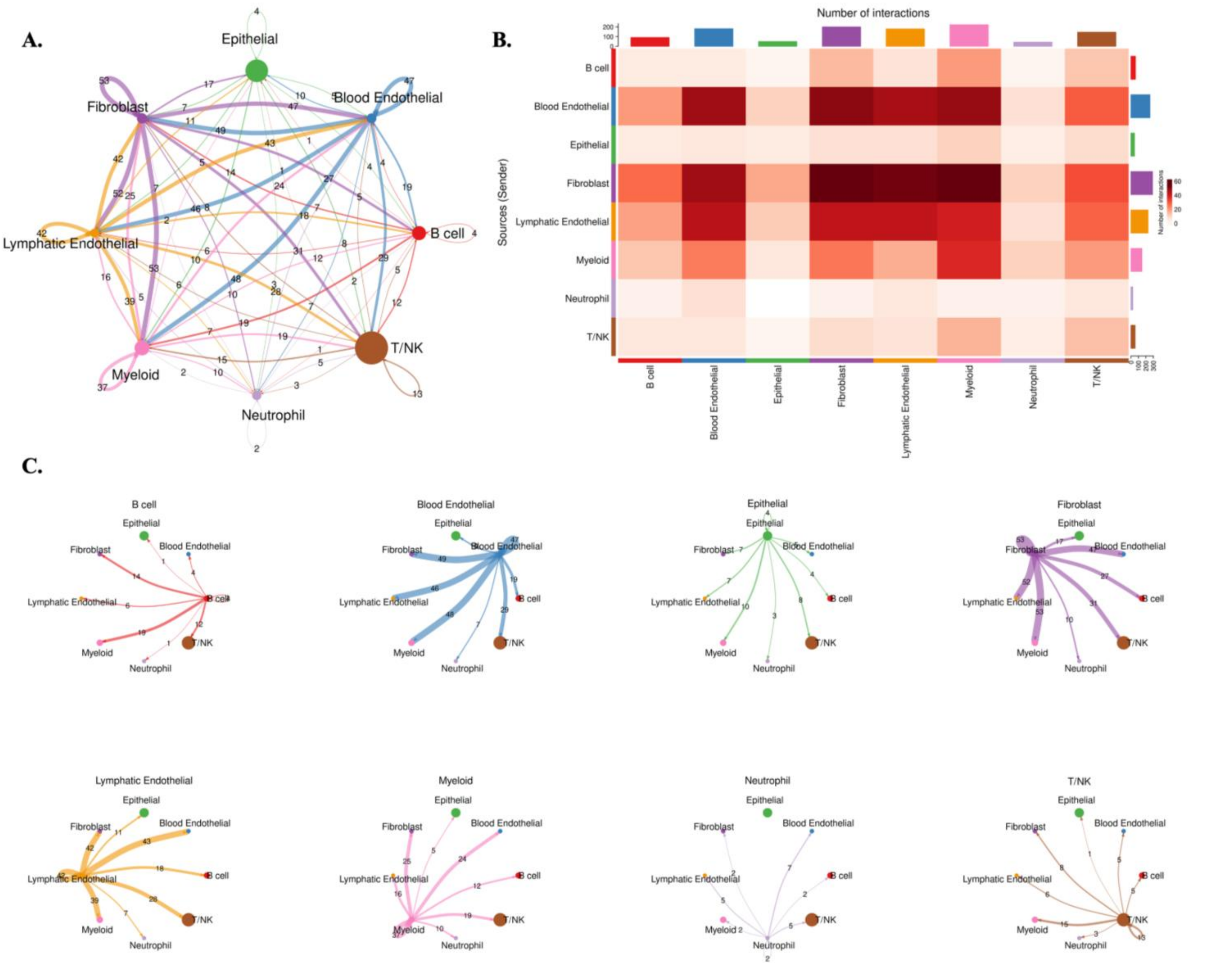

Supplementary Figure S11: Cell-cell communication networks in *AP1AR*-low lung tumor samples generated using a CellChat analysis. (A) Overall intercellular communication network among major cell lineages in *AP1AR*-low tumors. **(B)** Heatmap summarizing the total number of signaling interactions between sender and receiver cell populations. **(C)** Cell-type-specific network views illustrating directional communication from each major lineage.
